# Effect of quaternary ammonium surfactants on biomembranes using molecular dynamics simulation†

**DOI:** 10.1039/d3ra05030k

**Published:** 2023-11-09

**Authors:** Sedigheh Saddat Moosavi, Amin Reza Zolghadr

**Affiliations:** a Department of Chemistry, Shiraz University Shiraz 71946-84795 Iran arzolghadr@shirazu.ac.ir sedighehmusavi@yahoo.com +98 713 646 0788 +98 713 613 7157

## Abstract

Research conducted both prior to and after the emergence of the COVID-19 pandemic reveals a notable rise in human exposure to cleaning products, hand sanitizers, and personal care items. Moreover, there has been a corresponding increase in the environmental release of these chemicals. Cleaning and disinfecting products often contain quaternary ammonium compounds (QACs) with alkyl chains as long as 8–12 carbon atoms. The attachment of quaternary ammonium surfactants to the membrane resulted in the deformation of the bilayer and membrane disruption. Before interactions with cell membranes, these surfactant molecules may form different aggregates depending on their architecture. Interaction of surfactant monomers or clusters with the cell membrane changes the physiochemical properties of the biomembranes. To investigate this interaction and its influence on membrane properties, we conducted molecular dynamics simulations of cationic quaternary ammonium surfactants interacting with dipalmitoylphosphatidylcholine (DPPC) membranes. Our simulations revealed significant interactions between the surfactants and the phospholipids, leading to substantial alterations in the structure of the bilayer. The results are compared with the simulated anionic (SDS) and nonionic surfactants/bilayer systems. Various aspects were considered, including the aggregation process, migration behavior, and eventual equilibrium of these molecules at the interface between the membrane and water. This analysis used various techniques such as density profiles, distribution functions, cluster analysis, order parameters, hydrogen bonding (H-bonding), and mean-square displacements. The results indicate that while surfactants with shorter alkyl tails (*N*-(2-hydroxyethyl)-*N*,*N*-dimethyloctan-1-aminium chloride (HEDMOAC)) make strong hydrogen bonds with the phosphate group and ester oxygen of the phosphatidylcholine bilayer and enter toward the bilayer in the monomer form, surfactants of longer alkyl tails aggregated on the membrane head-water interface and interact minimally with the head groups of the DPPC bilayer. For DDEDMEAC, a quaternary ammonium surfactant with a hydrophobic alkyl chain consisting of two decanoate groups, alteration of the structural and dynamical properties of the bilayer is expected to be governed by two different factors. First, the structural order of DPPC increases as surfactant aggregates interact with the membrane head group. Second, the decrease in the order of the bilayer occurs due to the insertion of surfactant monomers within the hydrophobic region of the bilayer. Strong interactions between constituents of tetraoctylammonium bromide (TOABr) and lipid head groups lead to a reduction in interlipid interactions and order, which further results in increased porosity of cellular membranes. Understanding the extent of these interactions plays a pivotal role in the toxicological assessment of these surfactants.

## Abstract 2

The interactions between different surfactants and phospholipid membranes are investigated by molecular dynamics simulation.

## Graphical abstract

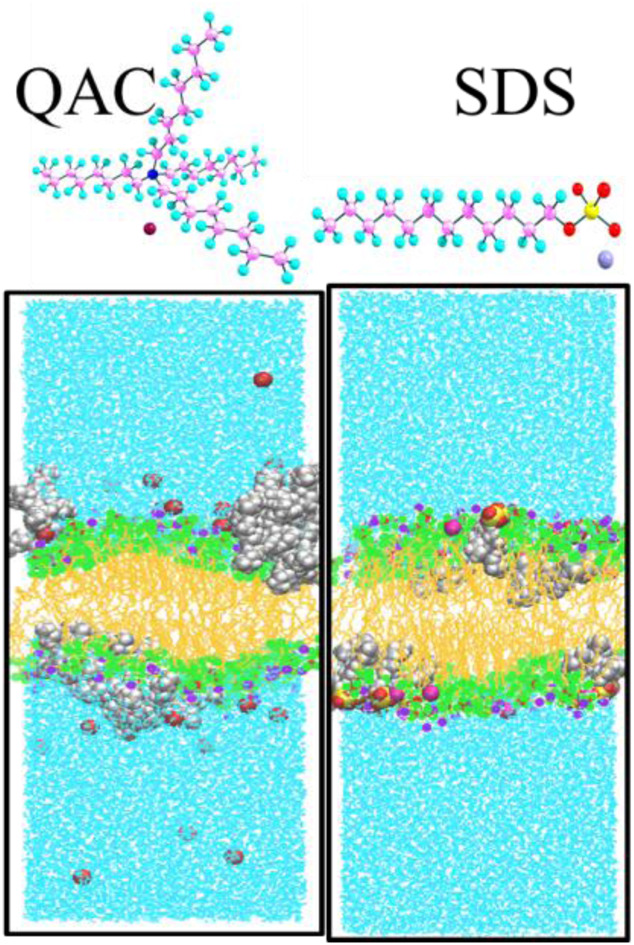

## 1. Introduction

Household cleaning compositions employ agents that consist of a diverse range of chemicals, including surface-active agents, phenols, and terpenoids. Surfactants, utilized not only in detergents but also in membrane biochemistry, play a crucial role in these compositions.^1^ The use of detergents leads to the release of surfactants into the environment, especially natural water bodies, raising essential questions about their interactions with biomembranes and their impact on membrane properties. These questions are of significant interest to both toxicology and environmental science.^2^

Quaternary ammonium compounds (QACs) comprise a central ammonium group carrying a permanent positive charge, usually connected to alkyl and aromatic substituents. The specific properties of these substituents and the length of the alkyl chain play a significant role in influencing the functionality, effectiveness, environmental behavior, and toxicity of QACs. Cleaning and disinfecting products commonly incorporate QACs with shorter alkyl chains (ranging from C8 to C16), whereas personal care products may contain QACs with alkyl chains as long as 22 carbons.^3^ QACs serve a wide array of functions, primarily acting as antimicrobials, surfactants, preservatives, antistatic and softening agents, and dispersants. They are commonly present in cleaning products, hand sanitizers, personal care items, various types of wipes, and pesticidal products. Despite their widespread use and release into the environment, most QACs have not been subjected to thorough regulatory assessment regarding potential adverse effects on human and ecological health. Surprisingly, even basic parameters essential for evaluating their potential harm, such as quantitative data on usage and volumes, physicochemical properties, exposure, and toxicity, remain lacking for the majority of these compounds.^4^ The presence of permanent positive charges enables QACs to readily bind to negatively charged solids. This strong affinity of QACs for sorption to particles and solids significantly influences these processes. In our previous work, we conducted molecular dynamics simulations to thoroughly investigate the interactions between surfactant solutions and solid surfaces.^5^

QACs in wastewater originate from both industrial and residential/commercial sources, making significant contributions to their presence.^6^ Recent environmental assessments have revealed frequent detections of QACs in surface waters across Europe, Asia, and North America. Typically, the concentrations of individual QAC compounds in these waters remain below 1 μg L^−1^.^7^ Additionally, specific QACs have a tendency to adsorb onto airborne particles and dust. Zheng *et al.* conducted a study where they measured 19 types of QACs in residential dust collected both before and during the COVID-19 pandemic.^8^ During the pandemic, QACs were detected in more than 90% of the collected samples, with concentrations ranging from 1.95 to 531 μg g^−1^. The total QAC concentrations in these samples were notably higher compared to the samples collected before the pandemic. Lebouf *et al.* reported increased levels of specific QACs, reaching up to 5.31 μg m^−3^ in the air shortly after the application of a product containing QACs through spraying.^9^ Certain job roles, like medical equipment preparers, housekeepers, floor strippers/waxers, endoscopy technicians, nurses, and dental assistants, often report spending over an hour per shift using QAC-containing products.^10–12^

Biomembranes, composed primarily of phospholipids, are essential components surrounding cells and their organelles.^13^ Biological membranes act as barriers and support membrane proteins within cells. Low molecular weight hydrophobic materials, including surfactants, are known to partition into the hydrophobic region of the membrane, increasing its fluidity and potentially leading to membrane disruption, cell leakage, and cell death.^14^ Susceptibility of biomembrane structure towards amphiphiles, ionic liquids and zwitterionic liquids are recently investigated by experiments and simulations.^15,16^ Evidence suggests that nonionic surfactants can interact with lipid membranes by forming channels through the membrane. In contrast, adding cationic surfactants to lipid membranes can lead to the formation of holes.^17^ Given the frequent contact between surfactants and cell membranes resulting from detergent use, understanding the interactions between surfactants and cell membranes is of fundamental importance.^18^

The study of phospholipid/surfactant mixed bilayer systems is crucial not only for understanding biochemical processes such as membrane dissolution and protein extraction but also for gaining insights into the structure and dynamic properties of such complex systems. However, the distribution and behavior of these external molecules within the bilayer matrix have not been thoroughly investigated.^2,19^ To address this issue, direct simulations of lipid bilayers and their interaction with surfactants are needed.^20^ Surfactant solutions exhibit complex phase behavior depending on thermodynamic conditions and concentration. Understanding the self-assembly and structure of surfactants at the molecular level has been the subject of significant experimental, theoretical, and computational studies. While phenomenological and theoretical models have provided insights into the properties of surfactant solutions, obtaining detailed molecular insights from such models is challenging.^21–25^

A comprehensive understanding of the precise nature of interactions within mixed bilayers that include cationic gemini surfactants remains elusive. Research by Almeida and colleagues has revealed that the arrangement and organization of these mixed bilayers are directly influenced by the vertical positioning of gemini molecules, indicating a complex interplay of effects. It has been observed that gemini surfactants with longer tails lead to increased atom density at the center of the bilayer, whereas surfactants with shorter tails decrease the density in that region.^26^

Furthermore a series of all-atom molecular dynamic simulations was conducted to investigate the structure of membranes formed using two specific surfactants: hexadecyl trimethyl ammonium dodecylsulfate (HTMA-DS) and ditetradecyldimethylammonium chloride (DTDAC).^27^ The simulations revealed that the HTMA-DS bilayer exhibited tightly packed chains in its membrane core, resulting in a gel-like structure. This behavior was attributed to the strong electrostatic attractions between HTMA and DS molecules. In contrast, the DTDAC molecules in the bilayer displayed a fluid-like and disordered packing of hydrophobic chains. This disorder was primarily caused by the electrostatic repulsion between the charged head groups of DTDAC. This study investigated the impact of di-alkyl chain cationic surfactants on the molecular scale in cationic vesicles. The findings highlight the effectiveness of such surfactants creating cationic vesicles, providing valuable insights into their role at the molecular level.

Also, molecular dynamics (MD) simulations and the umbrella sampling (US) method was used to investigate the transfer behavior and changes in Gibbs free energy of a sodium lauryl ether sulfate surfactant with two ether groups (SLE2S) as it transitions from a micelle to a ceramide bilayer.^28^ The potential of mean force (PMF) profiles generated for the transfer of SLE2S surfactant from a micelle to bulk water and then to a ceramide or DMPC bilayer unveiled attractive interactions between SLE2S monomers and ceramide lipid molecules. These interactions involved head-based electrostatic and hydrogen-bonding interactions, as well as hydrocarbon-tail-based hydrophobic interactions. These interactions provided sufficient energy for the SLE2S surfactant to transfer from the micelle into the ceramide bilayer. Alberto *et al.* explored the effects of dicationic alkylammonium bromide gemini surfactants on DPPC liposomes.^29^ Their findings revealed that surfactants with shorter tails (12 carbons) caused a decrease in the overall order of the bilayer. In comparison, those with longer tails (16 and 18 carbons) led to the formation of more ordered structures. The impact on the lipid order across the bilayer was further studied using a detailed fluorescence anisotropy analysis. Among the shorter tail surfactants, those with longer spacers (6 and 10 carbons) were found to have a more pronounced disruptive effect on the membrane, particularly near the lipid polar heads.

**Fig. 1:**
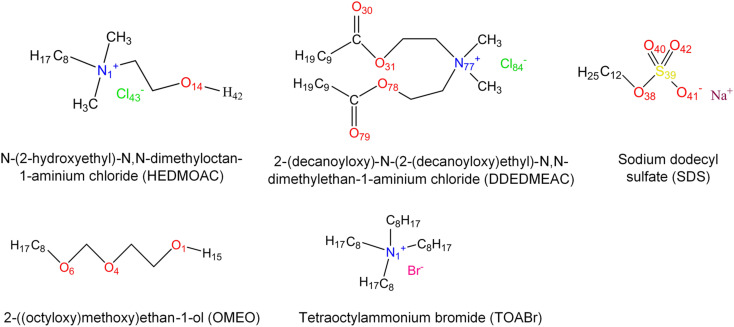
The molecular structure of surfactants with atom labeling.

The molecular aspects of self-assembly and interactions between anionic and cationic surfactant solutions at liquid/solid and liquid/membrane interfaces remain poorly understood. These interactions have significant implications for human health, as long-term exposure to these compounds could lead to toxic effects.^30,31^ In this study, our focus is on simulating a model membrane and exploring the interactions between the lipid layer and cationic, anionic, and neutral surfactants (see Fig. 1) by all-atom molecular dynamics (MD) simulations. Different lipid compounds can exert varying effects on membrane functionality. Notably, phospholipids, which encompass the choline group, form the predominant class of lipids found in eukaryotic cells.^32^ We selected a DPPC (1,2-dipalmitoyl-*sn*-glycero-3-phosphocholine) lipid bilayer as model membrane. These phospholipids are widely utilized in molecular dynamics (MD) simulations as they closely resemble naturally occurring phospholipids. Typically, these phospholipids consist of saturated *sn*-1 and unsaturated *sn*-2 acyl chains.^33^

To address this objective, three quaternary ammonium based surfactants of different natures are used as model cationic surfactants. *N*-(2-Hydroxyethyl)-*N*,*N*-dimethyloctan-1-aminium chloride (HEDMOAC) contains a hydrophobic alkyl chain comprising eight carbon atoms and a hydrophilic head group consisting of a dimethylammonium cation with an attached hydroxyethyl group. The chloride ion serves as the counterion to balance the charge of the surfactant. 2-(Ddecanoyloxy)-*N*-(2-(decanoyloxy)ethyl)-*N*,*N*-dimethylethan-1-aminium chloride (DDEDMEAC) is a quaternary ammonium surfactant with a hydrophobic alkyl chain consisting of two decanoyloxy (decanoate) groups and a hydrophilic head group composed of a dimethylammonium cation attached to an ethyl group. The chloride ion acts as the counterion to balance the charge of the surfactant. Another cationic surfactant that was used in this study is tetraoctylammonium bromide (TOABr), a quaternary ammonium surfactant with a hydrophobic alkyl chain composed of four octyl (C8) groups and a hydrophilic head group consisting of a tetraoctylammonium cation. The bromide ion serves as the counterion to balance the charge of the surfactant.^34,35^ Sodium dodecyl sulfate (SDS) is chosen as the representative anionic surfactant for comparison purposes, given its widespread use. It comprises a 12-carbon alkyl chain (dodecyl) attached to a sulfate group, with a sodium counterion. A model nonionic surfactant (OMEO) is also considered in this study. Their hydrophilic properties are attributed to multiple oxygen atoms in a specific region of the molecule. These oxygen atoms can form hydrogen bonds with water molecules, thus conferring hydrophilicity to the nonionic surfactants.

## 2. Computational methods

The structure optimization of each surfactant molecule was initially conducted using Gaussian 09 at the B3LYP/6-311++G** level. The atom charges for the surfactants were obtained through the utilization of the NBO method.^36^ For the simulation of lipids and surfactants, we employed the Gromacs software package (version 4.5.4)^37,38^ along with the GROMOS96 53a6 force field.^39^ To generate snapshots, visual molecular dynamics 1.9 (VMD) was utilized.^40^ In this study, we used pre-equilibrated coordinates and force field parameters of the DPPC bilayer, comprising 64 lipids per membrane leaflet. The primary coordinates for the DPPC lipid bilayer were obtained from the Tieleman group website.^41,42^ Water molecules were modeled using the SPC/E model.^43,44^ To enable a 2 × 10^−3^ ps time step, we implemented the LINKS algorithm.^45,46^ In all molecular dynamics (MD) simulations, a time step of 2 fs was utilized. The system was coupled to an isotropic pressure of 1 bar using the Berendsen barostat,^47^ with a time constant of 4 ps. To maintain a temperature of 310 K, the lipids, water, and surfactants were individually coupled to the thermostat using the velocity rescaling method,^48^ with a time constant of 0.1 ps. The temperature control was achieved using the Nose–Hoover thermostat.^49^ For the calculation of long-range electrostatic interactions, the particle mesh Ewald method was employed. The Lennard–Jones (LJ) pair potentials were evaluated within a cutoff distance of 1.2 nm, and a smooth switching function was applied above 1 nm. van der Waals interactions were cutoff with a switching function at a distance of 12 Å for all systems. To ensure the removal of any potential overlaps between water and surfactants, a minimization run was conducted for 500 ps in the NPT ensemble. This was followed by a dynamic simulation of 500 ps in the NVT ensemble, maintaining a temperature of 310 K.

For the construction of each system, a periodic box with dimensions of 6.5 × 6.5 × 18.5 nm^3^ was employed. To prepare the samples, a DPPC bilayer in contact with a water solution containing SDS, HEDMOAC, DDEDMEAC, TOABr, or OMEO surfactants was generated from a well-equilibrated configuration of neat DPPC. The lipid bilayer was placed at the center of the simulation box, and the initial configurations were created by adding 24 surfactant molecules on both sides of the simulation box. The remaining space within the box was filled with water molecules using standard GROMACS utilities. However, it should be noted that the water addition procedure employed by GROMACS led to slightly different numbers of water molecules in each case. Specifically, the SDS system contained 19 548 water molecules, while the HEDMOAC and DDEDMEAC systems had 18 328 and 18 266 water molecules, respectively. Although there are minor discrepancies in the number of water molecules, the concentration of surfactants in all samples remains close to 0.1 M. After the insertion of surfactant molecules, each system was subjected to an equilibration period of 10 ns to allow for the relaxation of the bilayer/surfactant systems. This extended equilibration time was necessary to attain a stationary distribution of ions throughout the system and to ensure the relaxation of the system's configuration. To monitor the convergence to equilibrium, running averages of density profiles for the different species, as well as thermodynamic (volume) and mechanical (stress) properties, were computed. Statistical data collection was then carried out over 200 ns to gather meaningful insights into the behavior and properties of the systems under study. The extended equilibration time and subsequent data collection enabled the analysis of the systems under steady-state conditions and ensured reliable and accurate results.

### 2.1 Analysis

The analysis involved the computation of statistical properties by averaging of simulation trajectories. To enhance the accuracy of our results and estimate the associated uncertainties, we implemented a block averaging technique for the calculation of standard errors. This method entailed dividing the simulation data into distinct, non-overlapping blocks, allowing us to obtain more reliable estimates of the mean values and their corresponding uncertainties.

#### 2.1.1 Density profiles

The spatial positioning and averaged orientation ordering of surfactants at the water/DPPC interface were determined by calculating density profiles along the *z*-axis. This analysis was performed using the g_density utility from the GROMACS software package. Density profiles of the DPPC polar headgroups, DPPC tails, water, and surfactants were generated throughout the simulation time for each system.

#### 2.1.2 Deuterium order parameters

To investigate the impact of surfactants on the membrane structure, the carbon-hydrogen (or carbon–deuterium) order parameters of the lipid tails were evaluated. These order parameters provide insight into the alignment of lipid tails and are commonly measured using experimental NMR techniques. The order parameter, referred to as *S*^CD^, was calculated using the formula 1/2〈(3cos^2^*θ* − 1)〉, where *θ* represents the angle between the C–H bond vector and the bilayer normal of the nth carbon atom of *sn*-1 and *sn*-2 chains. Averaging was performed over the molecules and simulation time was denoted by the angular brackets. The order parameter serves as a quantitative measure of the alignment of lipid tails. Typically, the values of −*S*^CD^(*n*) for lipid bilayers range from 0 to 0.5. A value of 0.5 indicates complete alignment of the lipid tail with the bilayer normal, while values close to zero indicate a random orientation. The GROMACS program g_order was utilized to define and calculate the deuterium order parameters.

#### 2.1.3 Radial distribution functions (RDF)

Radial distribution functions were computed to gain insights into the probability distribution of water and surfactant molecules away from the lipid bilayer. RDF analysis provides information about the spatial arrangement and interactions between these molecules.

#### 2.1.4 Mean square displacement (MSD)

To explore the relationship between structures and dynamics, we calculated the mean square displacement (MSD) of surfactants. The MSD analysis allows us to investigate the mobility and diffusion behavior of the surfactant molecules. By tracking the dynamic coordinates of the particles, the mean square displacements of surfactant molecules can be calculated, providing valuable information about their motion and behavior over interval (*τ*):
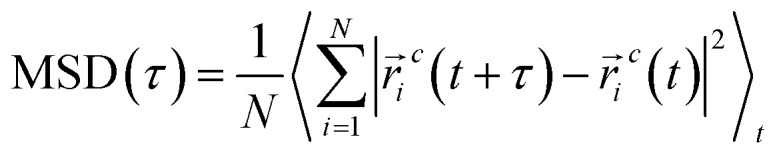
where *r⃑*_*i*_^*c*^ defines the coordinate of center of mass for particle *i* at time *t*. Also, the angular brackets show an ensemble average over time origins. The dynamics of the DPPC head group atoms normal to the membrane (*z*-direction) is excluded and MSDs are calculated parallel to the vector, *a⃑*, defined in the *xz*-plane (in the direction parallel to the membrane plane) by:
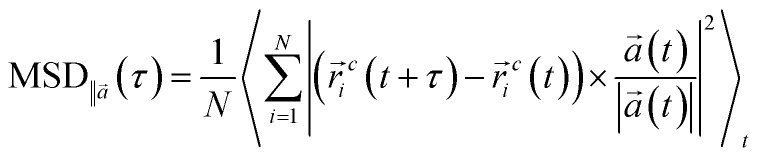


Diffusion coefficients of surfactant molecules, *D*_*i*_, were obtained from the linear regime of the 3D MSD curves at long simulation time using the Einstein relation,
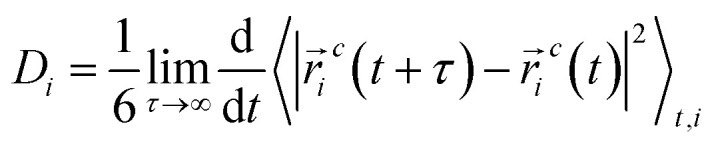


#### 2.1.5 Number of hydrogen bonds

We determined the average number of hydrogen bonds for DPPC, water, and surfactants along simulation time using g_hbond and a radius cut-off of 0.35 nm.

#### 2.1.6 Cluster analysis

The molecules were considered clusters if the distance of the closest approach was less than 3.5 Å. The cluster size *N̄*n is calculated by averaging the simulations trajectory from
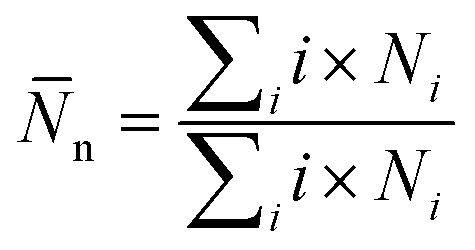
where *N*_*i*_ is the number of clusters, including *i* molecules. The sum in this equation runs from *i* = 2, *i.e.*, it does not account for monomers.

#### 2.1.7 Surface area per lipid

The average surface area per lipid (APL) was obtained by dividing the simulation cell area in the dimension parallel to the plane of the bilayer (*x*–*y* plane) by the number of lipids in one of the leaflets and averaging over all frames.

## 3. Results

In this study, we examine the mechanism by which surfactants can traverse a cell membrane, specifically through direct diffusion by employing atomistic simulations. The primary scientific question that motivated our research is: what is the nature of the interactions between surfactants and lipid membranes? By exploring this question, we seek to enhance our understanding of the fundamental structural and dynamical changes involved in the interaction between surfactants and lipid membranes.

**Fig. 2:**
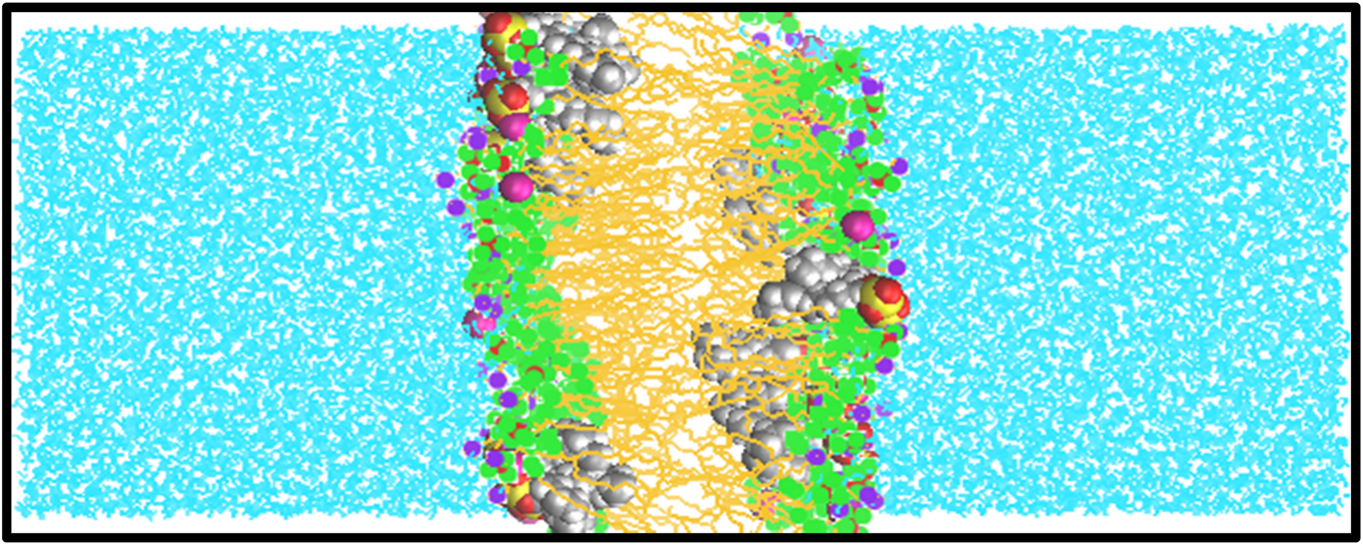
Snapshot of the bilayer system during MD simulations of SDS in DPPC/water after 200 ns of simulation. Water molecules are shown with blue lines. Tail and head groups of DPPC are represented in orange lines and green, blue, and brown points, respectively.

To the best of our knowledge, there have been limited theoretical studies addressing this specific problem. In this research, multiple simulations were conducted to determine the diffusivities of various surfactants within a membrane. Fig. 2 and S1–S5† present snapshots from the molecular dynamics (MD) simulations, illustrating a DPPC bilayer in contact with water solutions containing SDS, HEDMOAC, DDEDMEAC, TOABr, and OMEO surfactants. Each system was simulated for of 200 ns, allowing for a comprehensive analysis of their dynamics and behavior within the lipid bilayer.

Upon initiation of the simulation, all surfactants, except HEDMOAC, exhibited rapid accumulation within the water phase. These aggregated structures continued to interact with the membrane throughout the simulation timeframe. Specifically, in the case of SDS (as depicted in Fig. 2), the aggregates formed by both cations and anions became incorporated into the lipid bilayer. A detailed examination of the simulated snapshots, revealed that SDS aggregates within the bilayer tended to orient themselves in a manner where the polar head group atoms (*e.g.*, O_41_ atom) and cations (Na^+^ ions) remained in closer proximity to the polar region of the DPPC lipid molecules. Additionally, the terminal methyl groups on the alkyl chain of SDS molecules (*C*_Tail_) were solvated within the non-polar region of the bilayer.

The snapshot of the HEDMOAC system reveals a distinct situation compared to the other systems, primarily due to differences in structure and solubility limits among the compounds. In this case, the cations of HEDMOAC are incorporated into the lipid bilayer, while the Cl^−^ anions are adsorbed at the surface of the bilayer. This arrangement establishes an equilibrium state, where the bilayer-associated HEDMOAC cations maintain a coulombic attraction with the Cl^−^ anions dispersed in the surrounding water solution (see Fig. S2†). The stability of the anion film at the lipid/water interface is further enhanced by several factors. Firstly, the attractive interaction between the Cl^−^ anions and the polar head of DPPC, driven by strong coulombic forces. Additionally, the interactions between the Cl^−^ ions and the cationic part of the surfactant play a role in stabilizing the anion layer. In systems containing chloride anions, these ions tend to be distributed relatively evenly in both the water phase and the bilayer's head group region, primarily due to the high affinity of Cl^−^ for water. Overall, the behavior and distribution of ions in the HEDMOAC system demonstrate the intricate interplay between coulombic forces, solubility, and specific interactions with the lipid bilayer, ultimately influencing the stability and arrangement of the system.

The snapshots presented in Fig. S3† depict the behavior of DDEDMEAC surfactant molecules, which form several distinct clusters or blobs distributed relatively evenly in both the water phase and the bilayer. These clusters are in equilibrium with a population of chloride ions present in the surrounding solution. In this case, the penetration of ions into the bilayer is limited, which can be attributed to a couple of factors. Firstly, the reduced mobility of ions within the surfactant clusters or blobs compared to ions in the bulk solution hinders their penetration into the bilayer. Additionally, the stabilization of cations and anions within the surfactant droplets, facilitated by coulombic forces, acts as a counteracting force, diminishing the driving force for ion penetration. The presence of the surfactant clusters, their distribution, and the stabilization of ions within them play a crucial role in determining the behavior of ions in the system. The limited penetration of ions into the bilayer observed in the case of DDEDMEAC can be attributed to the interplay between the mobility of ions within the surfactant blobs and the attractive forces acting on the ions, resulting in a unique equilibrium distribution of ions between the surfactant clusters, the water phase, and the bilayer.

Based on the snapshots depicted in Fig. S4,† it can be observed that the alterations in the bilayer structure caused by the interaction with TOABr aggregates are relatively more pronounced compared to other systems. In the case of the non-ionic surfactant OMEO, a stable cluster is present in the water phase, and some isolated molecules have penetrated into the bilayer (see Fig. S5†). The confirmation and quantification of qualitative descriptions based on simulation snapshots are supported by several quantitative computations, including density profiles, deuterium order parameters, radial distribution functions, mean square displacements, surface area per lipid, cluster size analysis, and the number of hydrogen bonds. These calculations are mentioned in the following context.

### 3.1 Density profiles of systems

**Fig. 3:**
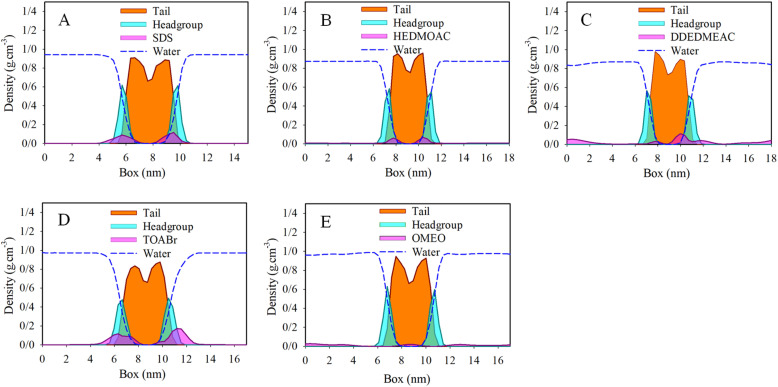
Total density profiles of (A) DPPC/SDS/water, (B) DPPC/HEDMOAC/water, (C) DPPC/DDEDMEAC/water, (D) DPPC/TOABr/water, and (E) DPPC/OMEO/water after 200 ns of simulation.

The results obtained from the molecular dynamics (MD) simulations are described by the mass density profiles along the *z*-axis, which is perpendicular to the surface. These density profiles are depicted in Fig. 3. The density profile of the lipid bilayer can be divided into two distinct regions: the headgroup region and the hydrocarbon chain region. In the present state, significant changes in the ordering of the hydrocarbon chains compared to the surfactant free control system were observed (Fig. S6†).^50^ The density profiles effectively capture the characteristic structure of the membrane. The mass density of the lipid tails has a depth near the middle of the bilayer, indicating the equilibrium distribution of the lipid bilayer. The density profiles provide further confirmation of the visual assessment regarding the equilibrium locations of the surfactants. Specifically, the density profiles demonstrate that all studied surfactant molecules have penetrated the membrane to a considerable extent, influencing the ordering of the membrane. Moreover, it appears that the surfactants form micelles in the water phase, which exhibit a preference for the interface layer. As the surfactants penetrate the membrane, the micelles become dissociated.

To gain further insights into the positioning of specific atoms within the surfactant and other constituents relative to the center of the membrane (see Fig. S7† for DPPC atoms labeling), we analyze the atomic density profiles. In panel (A) of Fig. S7,† the densities of selected atoms from both the lipid and SDS surfactant are depicted, along with the distribution of water oxygen atoms. The density profile illustrates that the terminal methyl group on the alkyl chain of SDS has penetrated into the membrane, toward the bilayer center. This observation suggests a distinct spatial distribution and orientation of the surfactant compared to the lipids in the membrane. Upon focusing on the polar region of the membrane, we observed that the phosphorus atoms in the lipid closely overlap with both oxygen and sulfur atoms of SDS surfactants.

Moving on to panel (B) of Fig. S7,† which corresponds to the analysis of HEDMOAC surfactants, we find that these surfactants do not penetrate deeply into the DPPC bilayer. Instead, they tend to reside closer to the water phase. The distribution of nitrogen HEDMOAC surfactants aligns with the distribution of membrane headgroups, exhibiting a shoulder in the respective distribution that overlaps with the polar groups of the phospholipids. Additionally, the terminal CH_3_ groups of HEDMOAC surfactants are partially penetrated the central region of the membrane.

In panel (C) of Fig. S7,† representing the DDEDMEAC surfactant, we observe that the oxygen atoms of the surfactant (for example, O_31_) have a distinct peak in the polar region of the bilayer. The terminal methyl groups of the surfactant clearly penetrate toward the hydrophibic region of DPPC. These analyses provide insights into the specific positioning and interactions of different surfactants with the lipid bilayer, highlighting the differences in their behavior and orientation relative to the membrane.

Panel (D) of Fig. S7† illustrates the analysis performed on the TOABr surfactant. In this case, the bromide ion of TOABr tends to dissolve in the water phase, while a peak corresponding to a monolayer formation (as described in the case of chloride anion) is observed at the water/bilayer interface. The nitrogen and also terminal methyl groups of the TOABr surfactant peaks are mainly concentrated in the headgroup region of the bilayer, which indicates a lower penetration tendency of this surfactant due to the large size cluster formation.

Now focusing on panel (E) of Fig. S7,† which pertains to the examination of the non-ionic OMEO surfactant, we can observe that the peak associated with the terminal methyl groups of OMEO surfactant aligns with the peak of the tail region of the DPPC membrane. This alignment indicates that the terminal methyl groups of the surfactant overlap with the hydrocarbon tail region of DPPC.

### 3.2 Deuterium order parameter

**Fig. 4:**
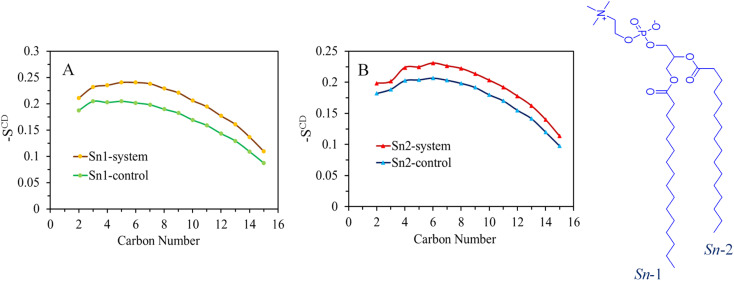
(A) Average values of the simulated *S*^CD^ in terms of the *sn*-1 and (B) *sn*-2 chains of lipid molecules in the DPPC model membrane over the 200 ns of the simulation for the SDS system. The calculated values of the control system are also shown.

The expansion of the membrane has a direct impact on the dynamics of the acyl chains that constitute the hydrophobic core of the bilayer. This effect is demonstrated by the order parameter data shown in Fig. 4 and S8–S11,† where excellent agreement is observed between the control system and the simulation results for the DPPC bilayer at a temperature of 310 K. The interactions between surfactants and lipids have a significant influence on the regulation of cell membrane structure and function. Lipids respond to the presence of surfactants by disrupting the order of the hydrophobic tails. Higher values of the order parameter indicate a more ordered structure, while lower values indicate a less ordered structure.

The deuterium order parameter *S*^CD^ for the lipid tails serves as an indicator of the orientation and arrangement of phospholipid tails within the bilayer relative to the bilayer's normal axis. It's important to note that an *S*^CD^ value of −0.5 signifies complete alignment of the lipid tail with the bilayer surface's normal axis. Conversely, a zero value denotes disorder, indicating the absence of any specific orientation within the system. In the chemical formula of the DPPC molecule shown on the right side of Fig. 4, the carbon tails are numbered accordingly: *sn*-1 chain consists of C_34_, C_36_–C_50_ and *sn*-2 of C_15_, C_17_–C_31_ (see the top panel of Fig. S7† for atom labeling). The order parameter profiles obtained for the pure DPPC system show that the orientation of order parameters is the same for both *sn*-1 and *sn*-2 chains. The control system, represented by the first area of relatively high lipid acyl chain order close to the interface, displays a plateau-like behavior (with some deviation at the beginning of the chain), followed by a second area where the order parameter gradually decreases towards lower *S*^CD^ values in the center of the membrane.

Upon comparing the order parameters of the control membrane without surfactants, it is evident that the systems containing SDS, TOABr, and OMEO exhibit higher values for both *sn*-1 and *sn*-2 chains. This increase in order indicates a more organized arrangement of lipid tails, which can be attributed to stronger van der Waals interactions between alkyl chains in the presence of surfactants. Notably, the order parameter of TOABr demonstrates a more significant increase compared to the other systems. Conversely, while the order parameter of the HEDMOAC system displays a substantial decrease relative to the control system, the system with DDEDMEAC experience a slight decrease.

### 3.3 Radial distribution function

To characterize the molecular structure of the studied systems, we analyzed the radial distribution function between the surfactants and other components. The RDF provides information about the probability of finding a particle at a certain radial distance (*r*) from a reference particle in a system of particles. For this particular analysis, we selected some atomic sites of surfactant molecules as reference sites, as shown in Fig. 1. Also, oxygen atoms of water (OW) and the oxygen (O_9_), carbon (C_31_), and phosphorus (P_8_) atoms of the lipid bilayer are selected to obtain the arrangement and interactions between these constituents.

**Fig. 5:**
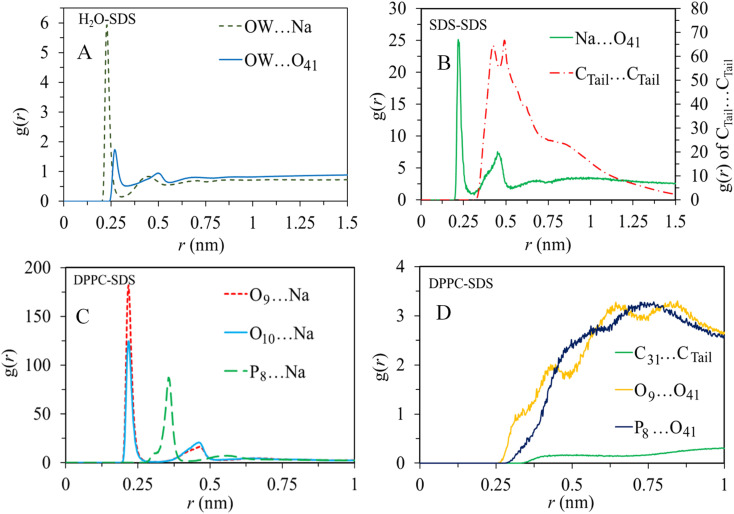
Radial distribution functions of (A) OW with cation and anion of SDS, (B) cation with the anion of SDS and tail of SDS, (C) cation of SDS with O_9_, O_10_, and P_8_ of DPPC, and (D) tail of SDS with tail of DPPC, and headgroups of DPPC with the anion of SDS.

The RDF analysis revealed exciting information about the interactions between different atoms in the system. In particular, we focused on the first peak values of the RDFs (Fig. 5) to examine the strength and probability of interactions. For the sodium (Na^+^) ions, it was observed that they have a higher probability of interacting with DPPC molecules compared to water molecules. Also, the interactions between (Na^+^) ions and the polar head group of SDS (O_41_) is more pronounced relative to its interaction with water. The correlations between alkyl tails of SDS molecules C_tail_⋯C_tail_ indicates substantial aggregation of surfactant molecules. These interactions are mainly driven by intermolecular van der Waals forces. While the correlations between alkyl tails of SDS and the hydrophobic region of phospholipid (C_31_) are less pronounced, the polar regions of SDS micelles interact substantially with the polar region of the lipid bilayer (P_8_⋯O_41_). Therefore, according to RDF analysis obtained by averaging trajectories over simulation time, SDS aggregates mainly interact with the polar region of the bilayer. On the other hand, the correlations between the surfactant polar region and water (O_41_⋯OW) first peak (almost at 0.25 nm) suggest a substantial tendency of SDS micelles toward the membrane/water interface.

The accumulation of SDS molecules at the bilayer/water interface primarily occurs because these molecules aggregate in the water phase, driven by intermolecular van der Waals interactions, as indicated by the computed radial distribution functions. Additionally, similar analyses were conducted for other surfactant molecules, specifically DDEDMEAC, TOABr, and OMEO, and the findings are presented in Fig. S12–S14.† The outcomes suggest comparable conclusions to those observed for SDS. In the case of DDEDMEAC, TOABr, and OMEO surfactants, the analysis demonstrated a significant propensity of surfactant molecules to interact, resulting in their aggregation within the water phase, which subsequently migrates towards the bilayer surface. The surfactant molecules primarily interact with the polar region of the bilayer.

In contrast, for HEDMOAC, as depicted in Fig. S15,† the radial distribution function analysis of C_tail_⋯C_tail_ interactions indicate a lower likelihood of interactions between surfactant molecules compared to the other surfactants investigated. Consequently, HEDMOAC does not exhibit aggregation at the examined concentration, and individual surfactant molecules tend to penetrate the lipid bilayer. Therefore, the probability of *C*_31_⋯*C*_tail_ correlations are relatively higher in this case. Moreover, there are significant interactions between the polar region of the bilayer and the polar head of the surfactant, as evidenced by the peak observed at approximately 0.26 nm in the O_9_⋯O_14_ correlation. In contrast, chloride anions primarily interact with water, as evident from the comparison between the OW⋯Cl_43_ peak and the P_8_⋯Cl_43_ peak.

To investigate the interactions between unsaturated lipids and surfactants, we performed simulations using ensemble of 1-palmitoyl-2-oleoylphosphatidylcholine (POPC) lipids, consisting of 256 POPC molecules. As the results was found to be unaffected by structure of phospholipids, we continued the simulation using the DPPC lipids (see Fig. S16–S18†). Semi-isotropic coupling is often used in membrane simulations to allow for different pressure scaling in the lateral (*XY*) and perpendicular (*Z*) directions to mimic realistic conditions. To check the pressure coupling effect, we performed some additional simulations for DPPC and POPC systems by using the semi-isotropic coupling. The results imply that the main outcomes of the interactions between surfactants and the membrane remain unaffected by the choice of pressure coupling (see Fig. S19–S21†).

### 3.4 Mean-squared displacements

**Fig. 6:**
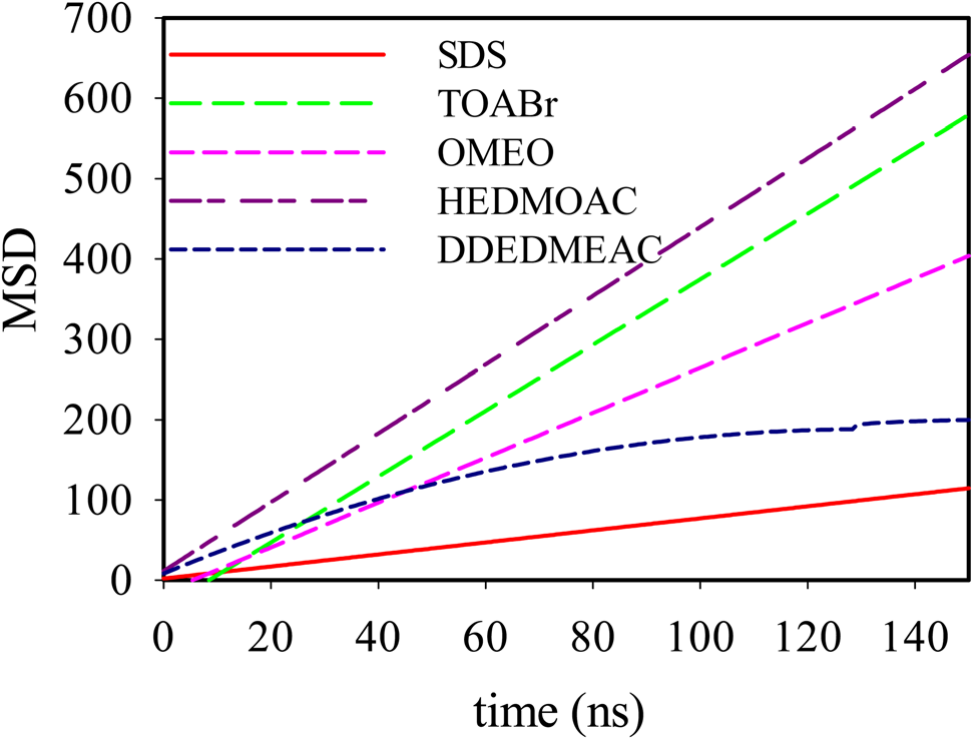
Mean-squared displacements of SDS, TOABr, DDEDMEAC, HEDMOAC, and OMEO molecules present at the water/DPPC interface.

The mean square displacement (MSD) analysis is utilized to investigate the molecular mobility of surfactants over time, particularly concerning their interaction with the membrane. In this study, the MSD of surfactants was measured based on their center of mass. A higher slope in the MSD curves indicates greater molecular mobility. Fig. 6 depicts the MSDs of surfactants in the DPPC-water interfacial model. The diffusion coefficient, *D*, is directly related to the slope of the mean square displacement through the Einstein relation. The Einstein relation assumes a linear dependence of the MSD on time for the determination of *D*. The lateral diffusion coefficients, *D*, calculated from the data shown in Fig. 6, are provided in Table S6.† The depicted MSDs in Fig. 6 exhibit distinct behaviors across three different time scales. To differentiate these time scales, one can calculate the *β* exponent over a range of time scales by employing the following equation
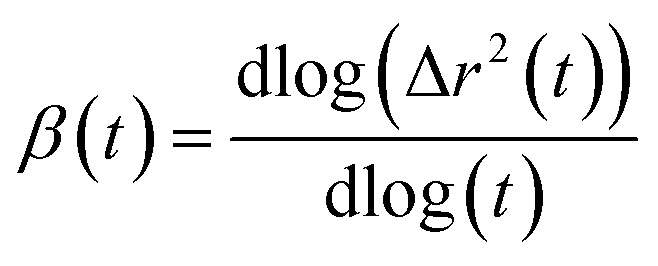
In the brief time intervals, the ion motions resemble near-ballistic trajectories, resulting in a *β* value of approximately 2. Conversely, over extended periods, the systems are expected to display typical linear diffusive behavior, where molecules have engaged in numerous collisions, yielding a *β* value of approximately 1. During the intermediate time span, the motion exhibits subdiffusive dynamics akin to those observed in supercooled liquids, often associated with cage escape phenomena, leading to a *β* value that is less than 1.

The trend in the slope of the MSD plots, from highest to lowest, corresponds to HEDMOAC > TOABr > OMEO > DDEDMEAC > SDS. This trend suggests that HEDMOAC molecules demonstrate higher diffusion rates than the other surfactant molecules, potentially attributable to their reduced tendency for accumulation. Conversely, the self-diffusion of SDS molecules is significantly slower than the other surfactants. Also, the diffusion coefficient of phospholipids, measured in our simulations by the mean square displacement of P8 atoms in the DPPC head is in the order of DDEDMEAC > SDS > OMEO > HEDMOAC > TOABr > OMEO (Fig. S22†).

### 3.5 Cluster analysis

The cluster sizes of surfactants were determined and presented in Table S7† based on the 200 ns simulation. The average cluster sizes throughout the simulation are depicted in Fig. S23.† It is evident from the simulations that the cluster sizes do not reach a maximum limit due to the interactions between surfactants and the lipid bilayer. Our investigation into cluster sizes provided further insights into the aggregation behavior of surfactants in the vicinity of the lipid bilayer. Notably, we observed that the surfactant TOABr, featuring four octyl branches, exhibited the largest aggregation numbers. As illustrated in Fig. S23,† there is a clear correlation between the size of the tail group and the preferred aggregation number. This indicates that larger tail groups tend to facilitate the formation of larger micellar cluster.

### 3.6 The average numbers of hydrogen bonds

Fig. S24† illustrates the average number of hydrogen bonds between various components, as determined using the “g_hbond” utility of GROMACS. During the simulation from 100 to 150 ns, the average number of H-bonds between SDS molecules and water is 85 ± 8.5. However, SDS molecules do not form H-bonds with each other or the bilayer. For the HEDMOAC molecules, Fig. S24B† shows the following average H-bond counts: HEDMOAC⋯Water: 28 ± 9, HEDMOAC⋯bilayer: 15 ± 8, and HEDMOAC⋯HEDMOAC: 2 ± 0.5. Regarding DDEDMEAC molecules, Fig. S24C† indicates an average H-bond count of 25 ± 8 with water. On the other hand, OMEO forms H-bonds with water (40 ± 20), the bilayer (2 ± 0.5), and with other OMEO molecules (4 ± 1). Notably, TOABr molecules did not participate in any H-bond interactions during the simulation.

### 3.7 Average surface area per lipid

For all simulated systems, the APL headgroup was calculated and compared with control systems. The value of APL of control DPPC system (0.614 ± 0.012 nm^2^) agrees with previous simulation and experimental results.^51,52^ The average APL headgroup for SDS, HEDMOAC, DDEDMEAC, TOABr, and OMEO systems are 0.610 ± 0.010 nm^2^, 0.664 ± 0.008 nm^2^, 0.660 ± 0.01 nm^2^, 0.580 ± 0.014 nm^2^, and 0.618 ± 0.010 nm^2^, respectively. While the APL increase due to the incorporation of HEDMOAC and DDEDMEAC surfactants, a substantial decrease in the APL values for the TOABr system is observed due to the tilting of the lipid monolayers. The APL for SDS is only slightly lower than that of the control system, while for OMEO, it is slightly higher than the control. This difference is attributed to the vdW interactions between DPPC leaflets and OMEO, along with the electrostatic interactions between the bilayer headgroup and SDS aggregates. The changes in APL are influenced by two distinct factors. First, the APL decreases as surfactant aggregates interact with the membrane headgroup. Second, the APL increases due to the insertion of surfactant molecules within the hydrophobic region of the bilayer. The direction of these effects depends on the ordering of the lipid chains.

## 4. Discussion

In this study, we have examined various cationic quaternary ammonium compounds, categorizing them into three distinct types based on their molecular structures. These types include HEDMOAC, characterized by a single octyl chain; DDEDMEAC, which features double decanoate tails; and TOABr, composed of four octyl chains. To provide a comprehensive comparison, we have also investigated anionic surfactants (SDS) and nonionic surfactants (OMEO). Our analysis reveals that quaternary ammonium surfactants with relatively small alkyl tails, such as HEDMOAC, tend to preferentially localize within the hydrophobic region of the bilayer. Density profiles of this system demonstrate the classical hydrophobic effect, resulting in low solubility in water but high solubility within the acyl chain region of the bilayer. Moreover, these surfactants exhibit a preference for partitioning their hydrophilic head region into the dense and highly charged head-group region of the bilayer, as opposed to remaining in the aqueous phase.

Notably, there was no observed exclusion of HEDMOAC from the ordered chain region towards the center of the bilayer. This lack of exclusion can be attributed to the amphiphilic nature of the surfactants under investigation, which possess both polar and nonpolar regions. Consequently, the equilibrated structure and density profiles of these surfactants are rather intricate. Previous simulations conducted within our research group have demonstrated a distinct tendency for certain molecules, such as carbazole derivatives and HSP90 inhibitors,^50,53,54^ to accumulate preferentially at the interface between the model membrane and the aqueous phase. Many of these studies attribute this preference for the interfacial region to the concurrent solvation of both the hydrophobic and hydrophilic regions of the solute within the two adjacent regions of the bilayer.

The diverse array of aggregate shapes observed for each type of surfactant studied can be attributed to the unique interactions exhibited by each surfactant molecule with other surfactant molecules, water, and the lipid bilayer constituents. To gain insight into the intermolecular interactions among surfactant molecules, we can examine the radial distribution function and analyze cluster sizes. Initially, when these molecules reside in the bulk water, quite distant from the membrane, it is the hydrophobic interactions involving the alkyl tails of surfactants (namely SDS, DDEDMEAC, TOABr, and OMEO) that are most likely to give rise to prominent peaks in the radial distribution functions. Consequently, in the aqueous phase, aggregates of varying sizes are formed for these surfactants.

The orientational preferences of these aggregates differ depending on their proximity to the bilayer. For instance, in the case of SDS, there is an interaction between the head group region and the surrounding water. On the other hand, when it comes to the DDEDMEAC surfactants, the head groups appear to be shielded from the surrounding water by lipid head group atoms, particularly their hydrophobic components. Similarly, the most probable RDF peaks observed between DPPC head groups and TOABr surfactant (Fig. S13†) suggest that quaternary ammonium cations are protected from the surrounding water molecules by the nearby phosphatidylcholine groups.

The critical micelle concentration (cmc) signifies the concentration at which surfactant molecules undergo self-assembly, forming micellar clusters in which the solvophobic groups are directed toward the core while the solvophilic groups remain at the interface. It is a well-known fact that the cmc decreases exponentially as the length of the tail group increases. Alterations in the architecture of surfactant molecules typically result in changes to the cmc and the sizes of the aggregates formed. This influence of surfactant architecture on micelle structure extends to the interactions between micelles and free aggregates, ultimately affecting the macroscopic properties of the systems. Among the surfactants we investigated, the cmc of only SDS has been reported in the literature, approximately at 0.01 M.^55^ To the best of our knowledge, while the concentration of surfactants in all our simulations was approximately 0.1 M, differences in the cmc of the surfactants we studied exert a notable influence on their aggregation states and, consequently, their interactions with cell membranes. According to our cluster size analysis, the surfactant TOABr, which boasts four octyl branches, exhibited the largest aggregation number. As illustrated in Fig. S23,† this suggests that the size of the tail group directly correlates with the preferred aggregation number, leading to a larger micelles within the system. Furthermore, the cluster size analysis highlights the role of surfactant architecture in the formation of aggregates. The larger aggregation numbers observed for surfactants with bulkier tail groups, such as TOABr, suggest that tail group size can influence the size and stability of surfactant aggregates in the membrane environment.

We initially determined the diffusion coefficients for each surfactant by conducting linear fits to the slopes of the MSD(*t*) functions, focusing on the subdiffusive region of the data. Notably, within all quaternary ammonium surfactants, it was observed that the anions exhibited faster motion compared to the cations. The self-diffusion coefficients values obtained for both the anions and cations of the surfactants are in the order of 10^−10^ m^2^ s^−1^. The analysis of MSD, as shown in Fig. 6, provides valuable information regarding the dynamics of surfactant molecules in proximity to the lipid bilayer. Notably, we observed distinct behaviors in MSD across different time scales. At short timescales, the motion of ions appeared nearly ballistic, characterized by an exponent (*β*) of approximately 2. This behavior suggests rapid and relatively unimpeded motion, indicative of surfactant molecules exploring their surroundings freely. Conversely, at longer timescales, the systems exhibited normal linear diffusive behavior, with *β* approaching 1. This regime corresponds to a more predictable motion where surfactant molecules have undergone multiple collisions, resulting in a smoother and more diffusive trajectory. However, the most intriguing results were observed in the intermediate-time range. Here, the motion displayed subdiffusive dynamics (*β* < 1), resembling the behavior often observed in supercooled liquids. This subdiffusive behavior can be linked to phenomena such as cage escape, where surfactant molecules experience restrictions in their movement due to interactions with the surrounding lipid environment.

Comparing the mean squared displacement of surfactants with the motion of the P8 atom within the bilayer, it becomes evident that surfactants do not replicate the behavior of lipid P8. Several factors come into play in influencing the dynamics of the P8 atom within the DPPC bilayer in our studied system. One notable factor is the substantial aggregation of TOABr, which results in heightened interactions with DPPC. This interaction has the effect of significantly increasing the order and surface pressure of the lipid. Consequently, the condensing and ordering influence exerted by TOABr on the lipid bilayer leads to a reduction in the diffusion of the lipid P8 atom.

## 5. Conclusions

We employed molecular dynamics simulations based on a classical empirical force field to investigate the interaction between DPPC bilayer and various surfactant solutions including quaternary ammonium surfactants. Our simulation results reveal significant and specific interactions between ionic and nonionic surfactants with DPPC. In the case of SDS, Na^+^ ions are found to be involved in strong bonding with the oxygen atoms of DPPC head groups, and they mainly reside in the interfacial regions (according to the RDF results). In contrast, dodecyl sulfate ions get accumulated as micelles at the water-lipid interfacial region. The cationic surfactant that contains a hydrophobic alkyl chain comprising eight carbon atoms and a hydrophilic head group consisting of a dimethylammonium cation with an attached hydroxyethyl group of 8 alkyl chain length (HEDMOAC) does not exhibit any spatial aggregation on the membrane head-water interface. In this case, the incorporation of the hydrophobic alkyl chains of the surfactants into the lipid bilayer has the most pronounced effect on the structure and stability of DPPC. The positioning and orientation of these alkyl chains within the bilayer are optimized to ensure favorable solvation of the hydrophobic tails within the inner hydrocarbon layer of DPPC. The presence of HEDMOAC surfactants, leads to an increase in disorder within the DPPC bilayer. Additionally, this incorporation facilitates the screening of the polar region of the surfactants by the zwitterionic head of the phospholipid, contributing to the overall stability of the system. These findings emphasize the crucial role played by the hydrophobic alkyl chains of the surfactants in interacting with and modifying the structure of the DPPC bilayer. While the specific positioning and orientation of the DDEDMEAC surfactant aggregates and monomers within the bilayer optimize the interactions between the hydrophobic and polar regions, leading to the stability of the DPPC structure, TOABr aggregate destabilizes the bilayer structure. Our simulations also revealed changes in the diffusion coefficient of phospholipids, as indicated by the mean square displacement of P8 atoms in the DPPC head. These changes suggest faster dynamics of the phospholipids upon absorption of the surfactants.

## Conflicts of interest

There are no conflicts to declare.

## Supplementary Material

RA-013-D3RA05030K-s001
