# Supplementary material for: Effect of quaternary ammonium surfactants on biomembranes using molecular dynamics simulation
Source: RSC Adv. 2023 Nov 9;13(47):33175–86. doi: 10.1039/d3ra05030k (PMC10634318; doi:10.1039/d3ra05030k)
Supplement: RA-013-D3RA05030K-s001 [file RA-013-D3RA05030K-s001.pdf]

Electronic Supplementary Material (ESI) for RSC Advances.
This journal is © The Royal Society of Chemistry 2023

Electronic Supplementary Information

Effect of Quaternary Ammonium Surfactants on Biomembranes using Molecular Dynamics Simulation

Sedigheh Saddat Moosavi, Amin Reza Zolghadr*

Department of Chemistry, Shiraz University, Shiraz, 71946-84795, Iran

*Corresponding author: arzolghadr@shirazu.ac.ir; Tel: +98 713 613 7157, Fax: +98 713 646 0788, ORCID: 0000-0002-6289-3794 (A.R.Z)

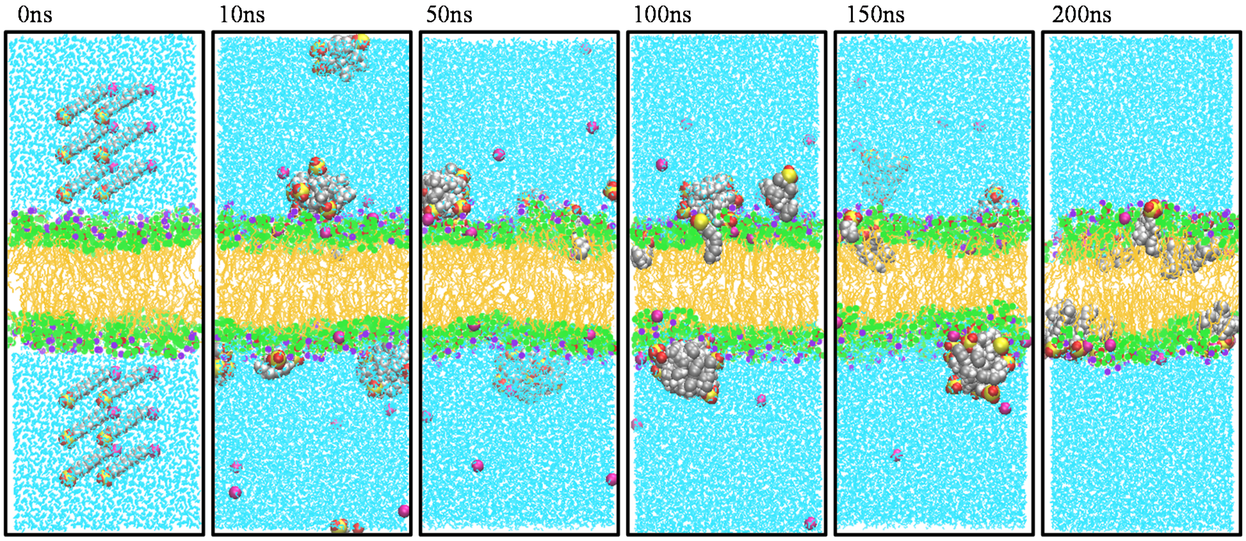

Fig. S1. Snapshots of the bilayer systems during MD simulations of SDS in DPPC/water after 200 ns of simulation. Water molecules are shown with blue lines. Tail and head groups of DPPC are represented in orange lines and green, blue and brown points, respectively.

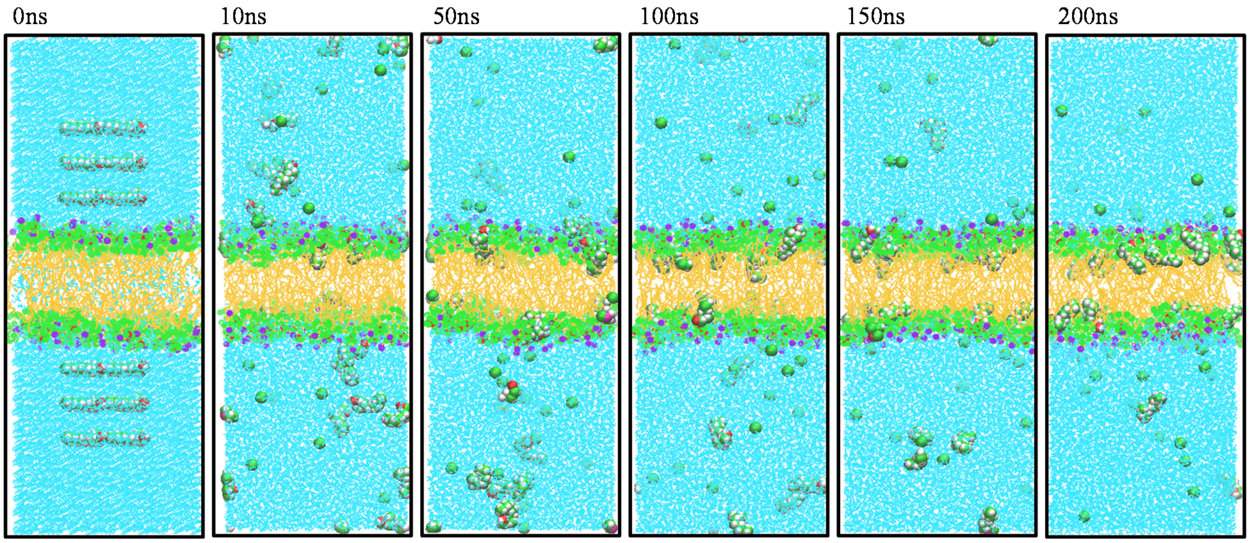

Fig. S2. MD simulation sample snapshots of the distribution of HEDMOAC in DPPC/water after 200 ns of simulation. Water molecules are shown with blue lines. Tail and head groups of DPPC are represented in orange lines and green, blue and brown points, respectively.

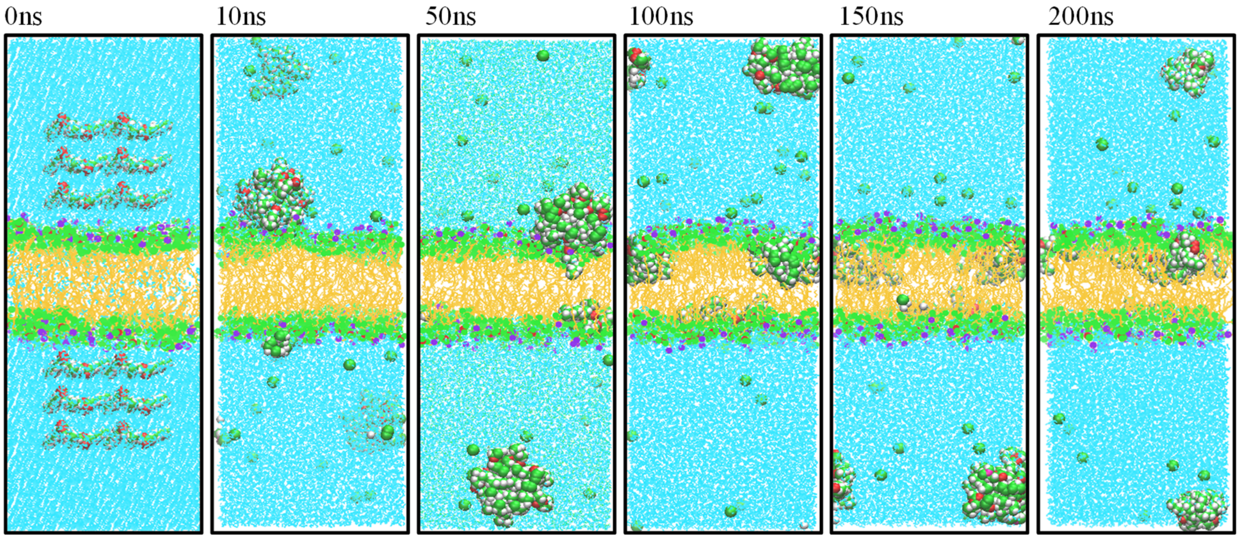

Fig. S3. MD simulation sample snapshots of the distribution of DDEDMEAC in DPPC/water after 200 ns of simulation. Water molecules are shown with blue lines. Tail and head groups of DPPC are represented in orange lines and green, blue and brown points, respectively.

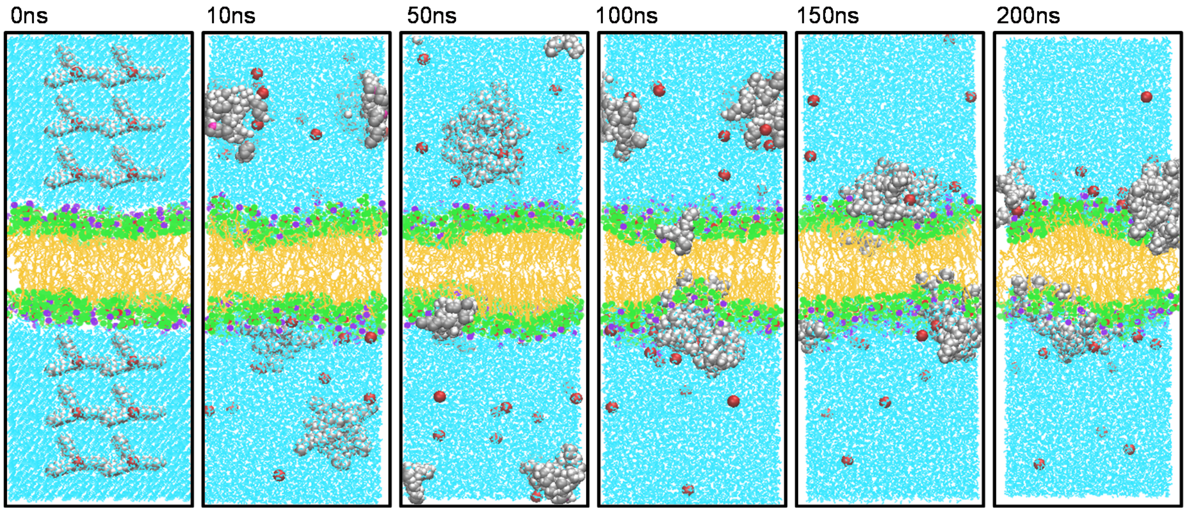

Fig. S4. MD simulation sample snapshots of the distribution of TOABr in DPPC/water after 200 ns of simulation. Water molecules are shown with blue lines. Tail and head groups of DPPC are represented in orange lines and green, blue and brown points, respectively.

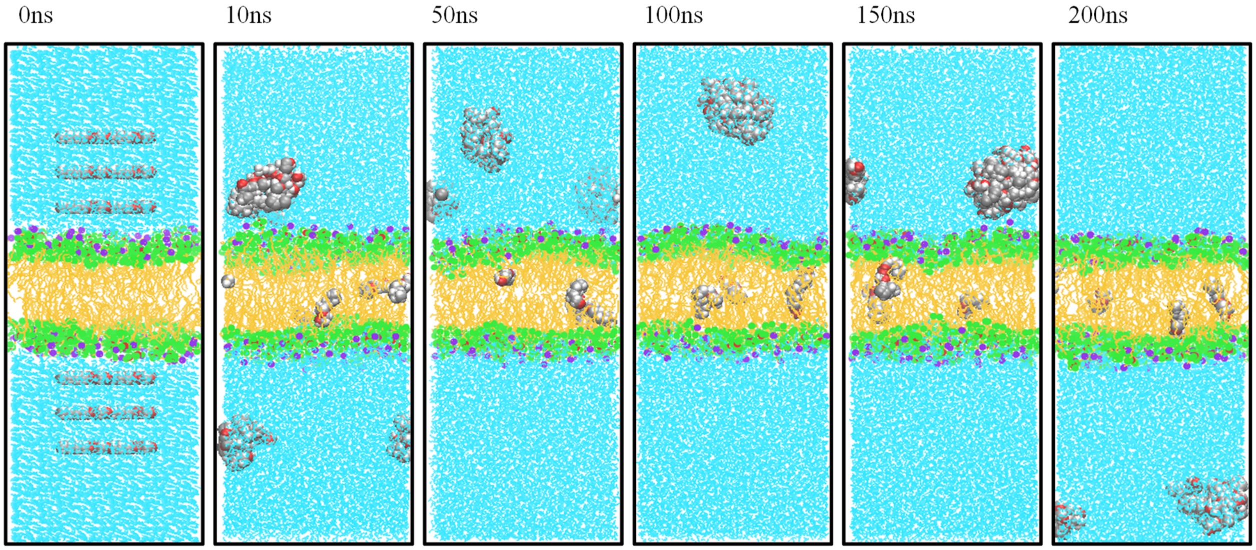

Fig. S5. MD simulation sample snapshots of the distribution of OMEO in DPPC/water after 200 ns of simulation. Water molecules are shown with blue lines. Tail and head groups of DPPC are represented in orange lines and green, blue and brown points, respectively.

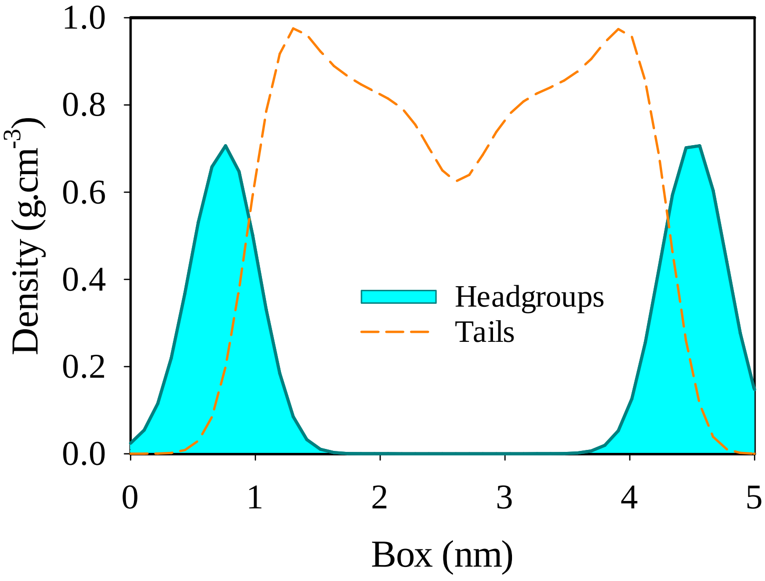

Fig. S6. Density profiles of the headgroups, the lipid tail obtained from the trajectories of the control system.[50]

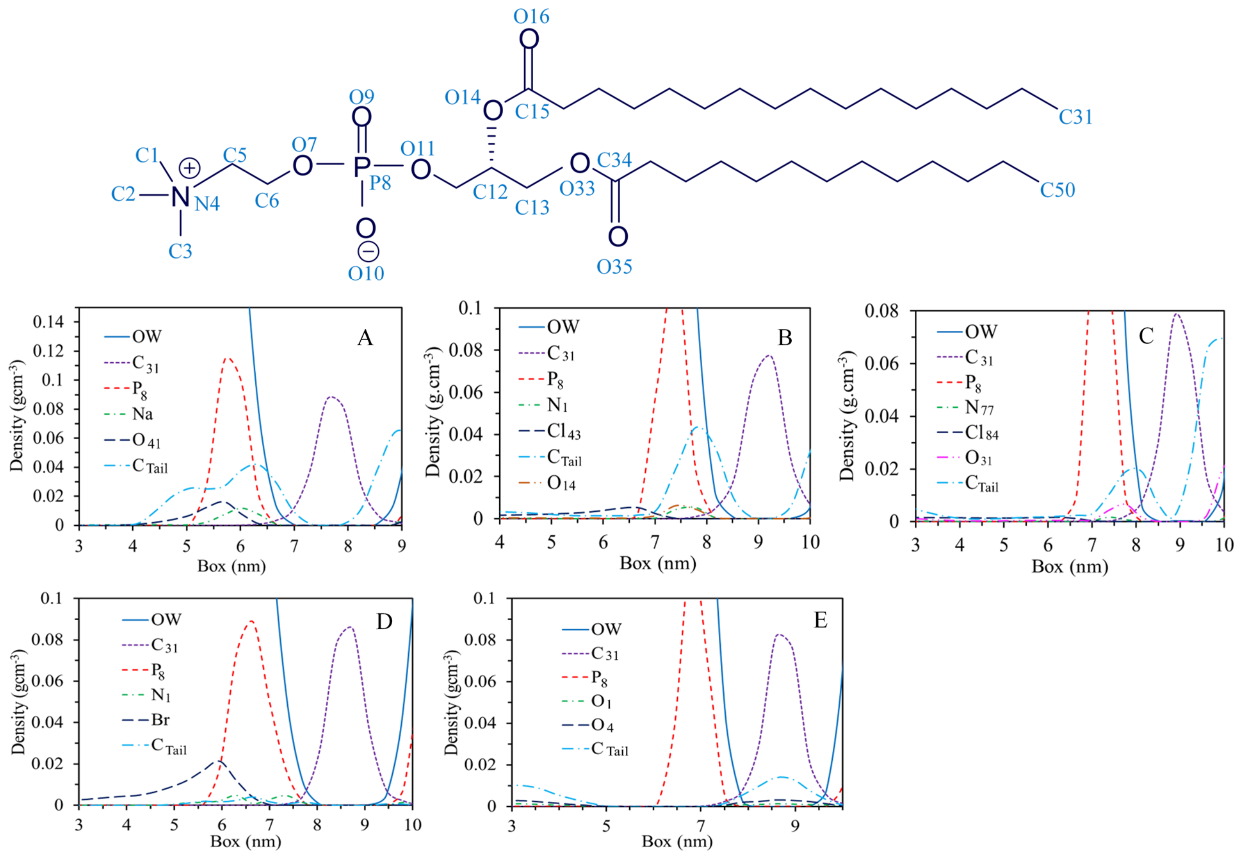

Fig. S7 Density profiles of specific atom/groups relative to the z-axis, perpendicular to the plane of the bilayer, exploited from the MD simulations performed at T = 310 K, for (A) DPPC/SDS (B) DPPC/HEDMOAC (C) DPPC/ DDEDMEAC (D) DPPC/TOABr and (E) DPPC/OMEO systems.

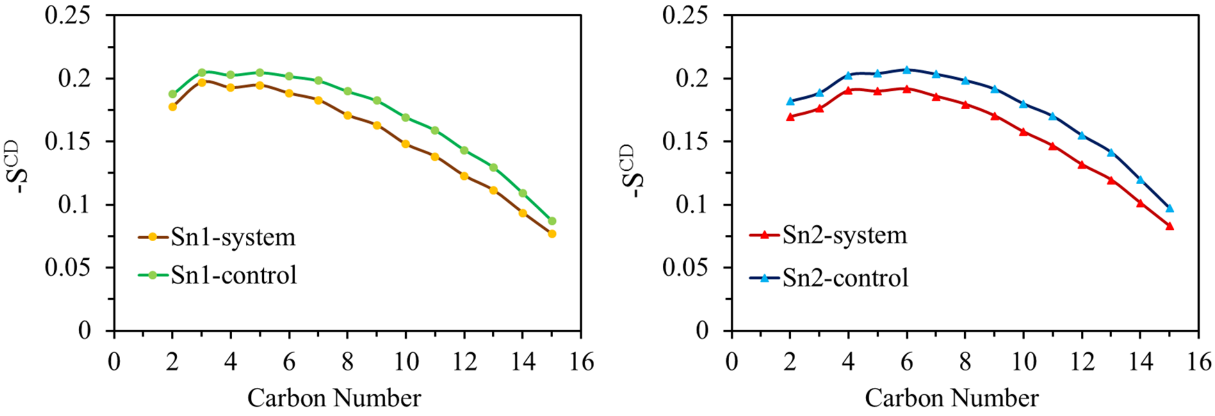

Fig. S8. (A) Average deuterium order parameters obtained for the sn-1 (B) and sn-2 chains of DPPC over the 150 ns of the simulation for HEDMOAC system. The calculated values of the control system are also shown.

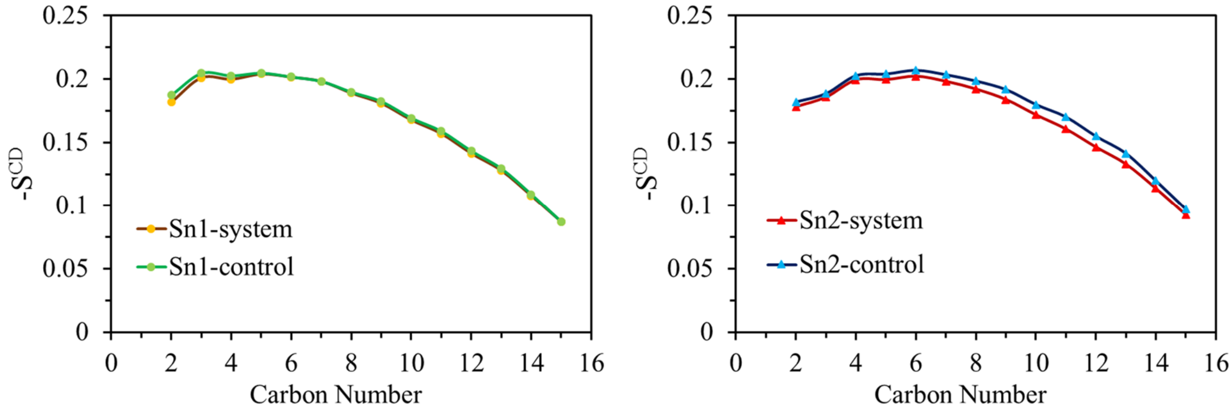

Fig. S9. (A) Average deuterium order parameters obtained for the sn-1 (B) and sn-2 chains of DPPC over the 150 ns of the simulation for DDEDMEAC system. The calculated values of the control system are also shown.

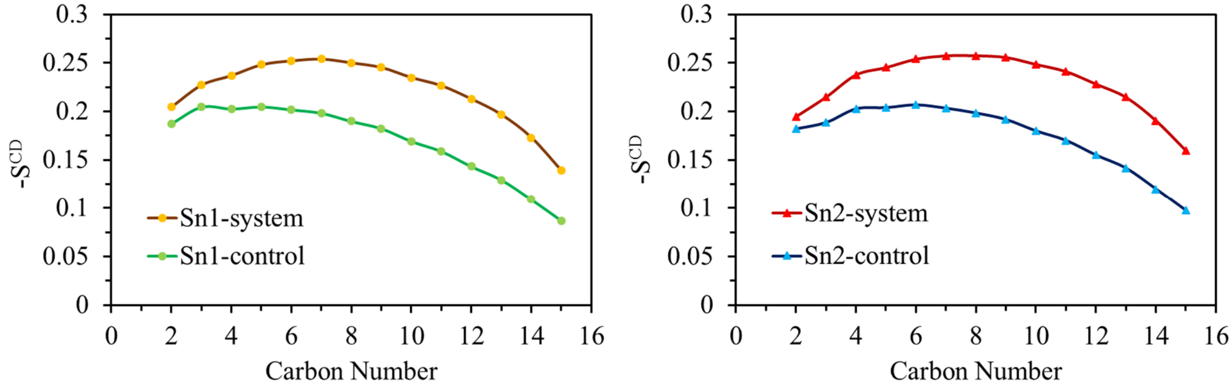

Fig. S10. (A) Average deuterium order parameters obtained for the sn-1 (B) and sn-2 chains of DPPC over the 150 ns of the simulation for TOABr system. The calculated values of the control system are also shown.

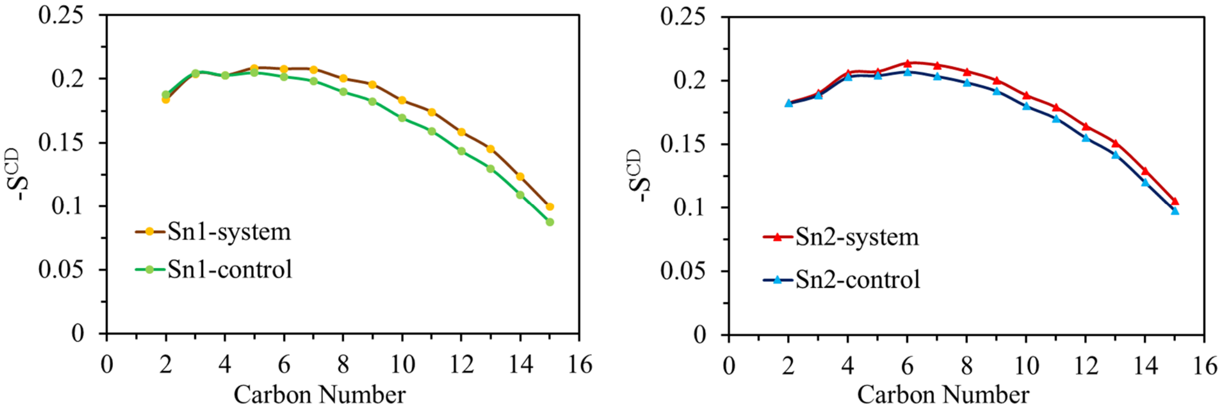

Fig. S11. (A) Average deuterium order parameters obtained for the sn-1 (B) and sn-2 chains of DPPC over the 150 ns of the simulation for OMEO system. The calculated values of the control system are also shown.

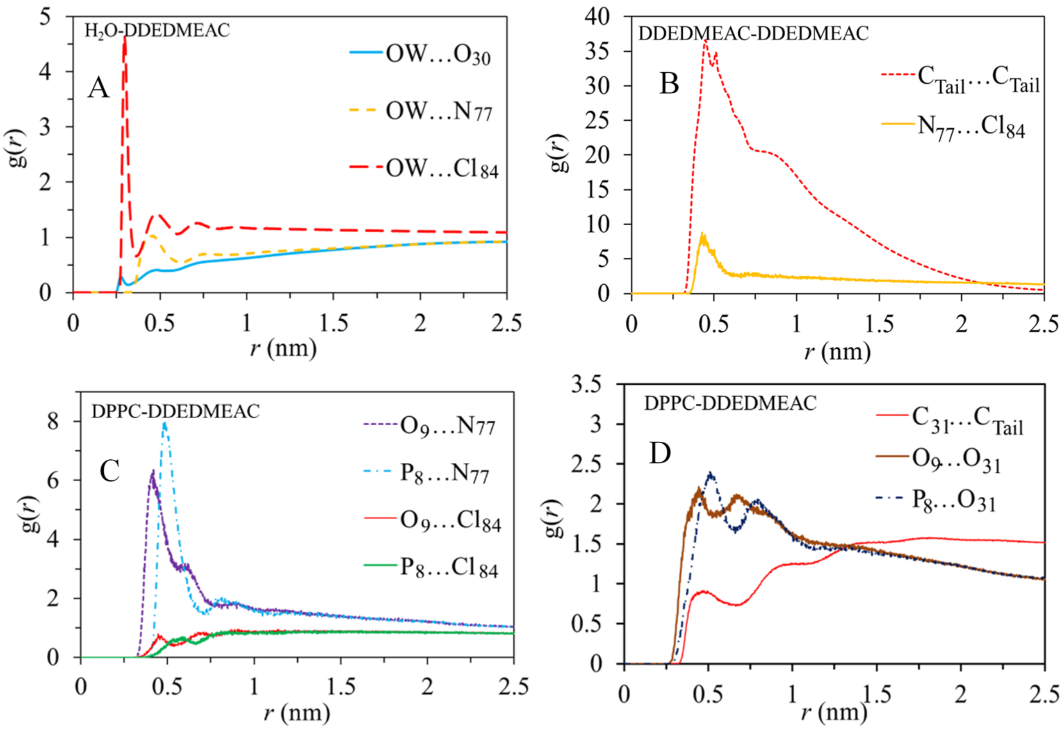

Fig. S12. Radial distribution functions of (A) OW with cation, anion and O_{30} of DDEDMEAC (B) cation with anion and tail of DDEDMEAC (C) cation and anion of DDEDMEAC with headgroups of DPPC and (D) O_{31} and tail of DDEDMEAC with headgroups and tail of DPPC.

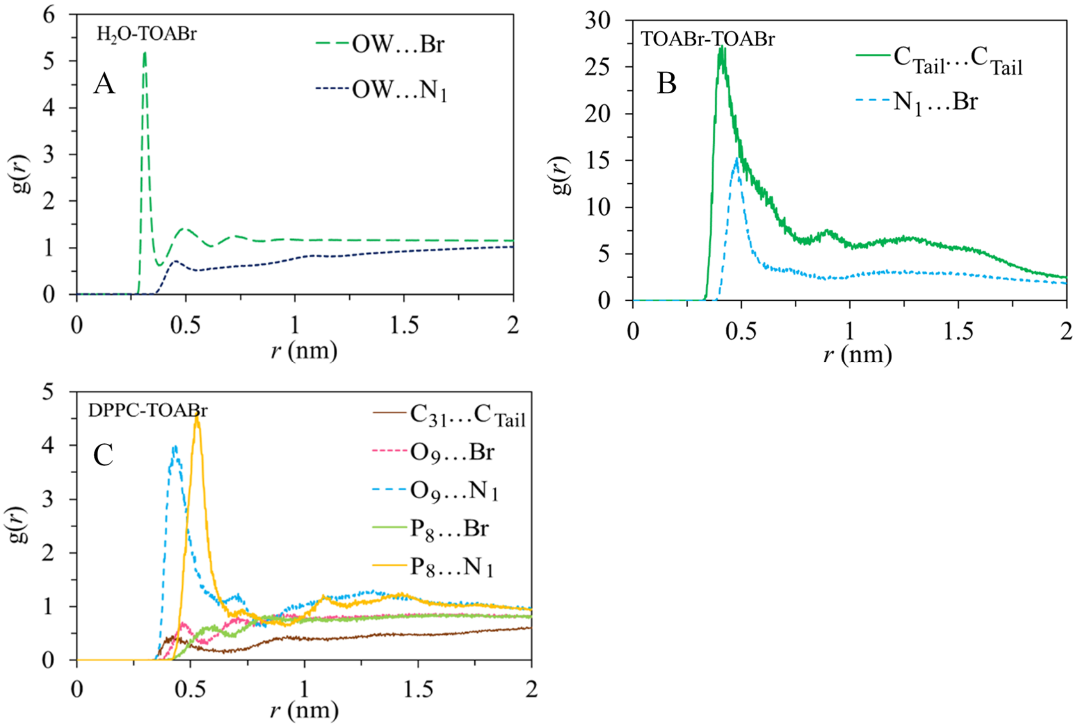

Fig. S13. Radial distribution functions of (A) OW with cation and anion of TOABr (B) cation with anion and tail of TOABr (C) cation, anion and tail TOABr with headgroups and tail of DPPC.

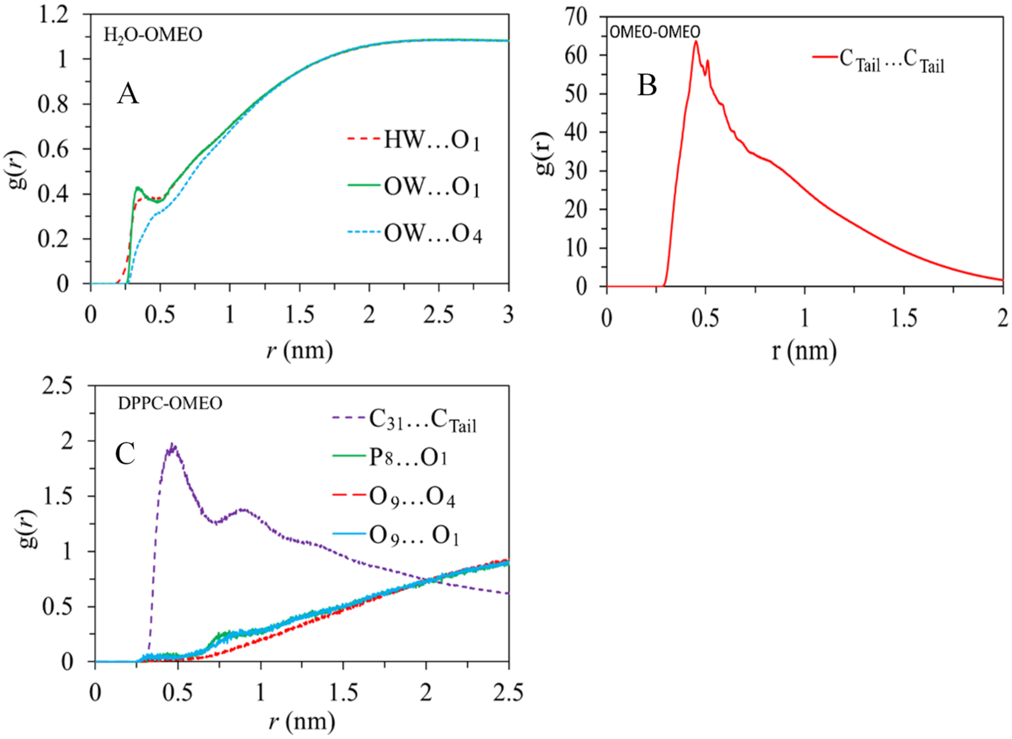

Fig. S14. Radial distribution functions of (A) OW and HW with O_1 and and O_4 of OMEO (B) tail of OMEO (C) headgroups and tail of DPPC with O_1, O_4 and tail of OMEO.

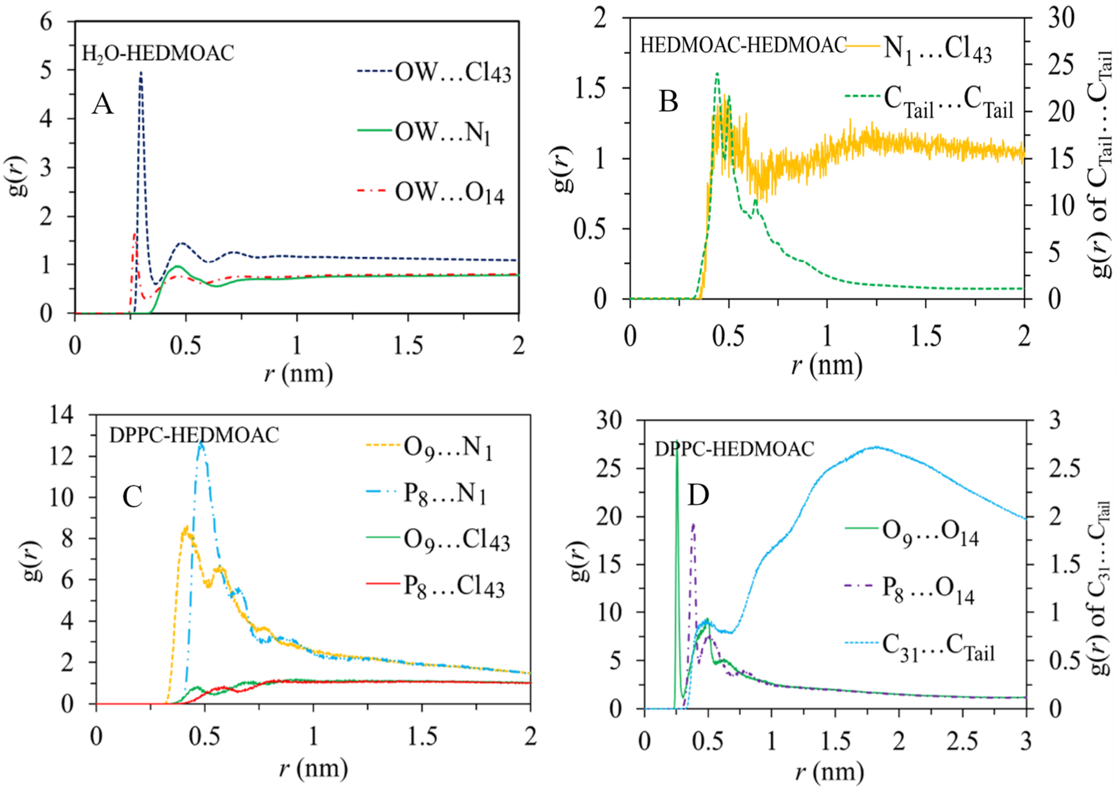

Fig. S15 Radial distribution functions of (A) OW with cation, anion, and O_{14} of HEDMOAC (B) cation with anion of HEDMOAC and tail of HEDMOAC (C) headgroups of DPPC with cation and anion of HEDMOAC (D) O14 of HEDMOAC with headgroups of DPPC and tail of DPPC with tail of HEDMOAC.

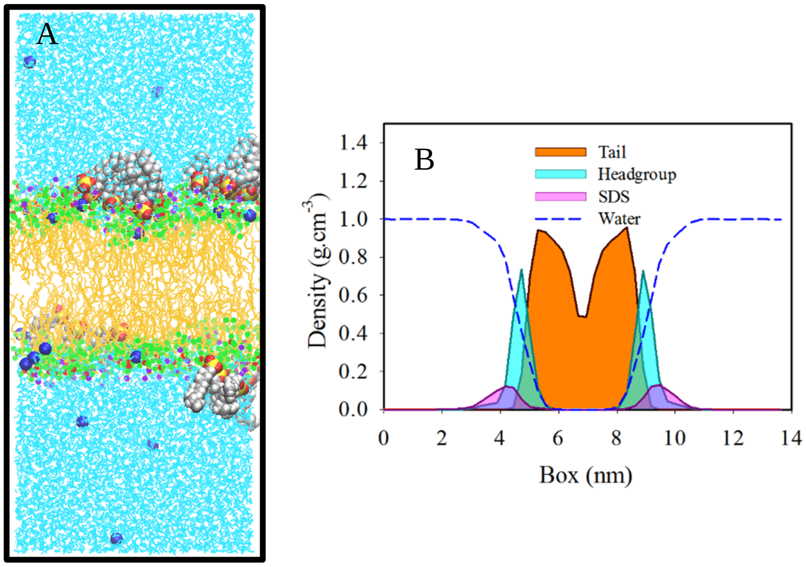

Fig. S16 (A) Snapshot of the bilayer system during MD simulations of SDS in POPC/water after 50 ns of simulation. (B) Total density profiles of POPC/SDS/water.

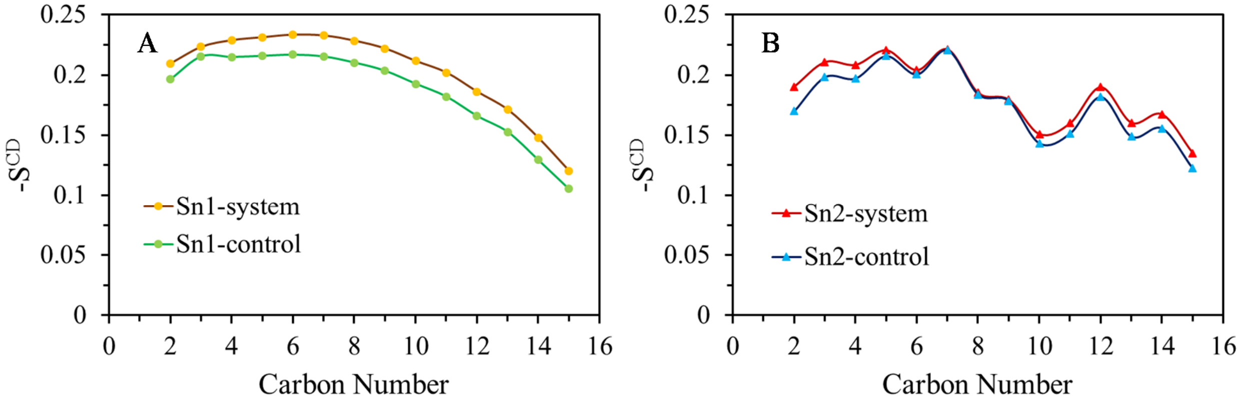

Fig. S17 (A) Average values of the simulated S^{CD} in terms of the sn-1 (B) and sn-2 chains of lipid molecules in the POPC model membrane over the 50 ns of the simulation for SDS system. The calculated values of the control system are also shown.

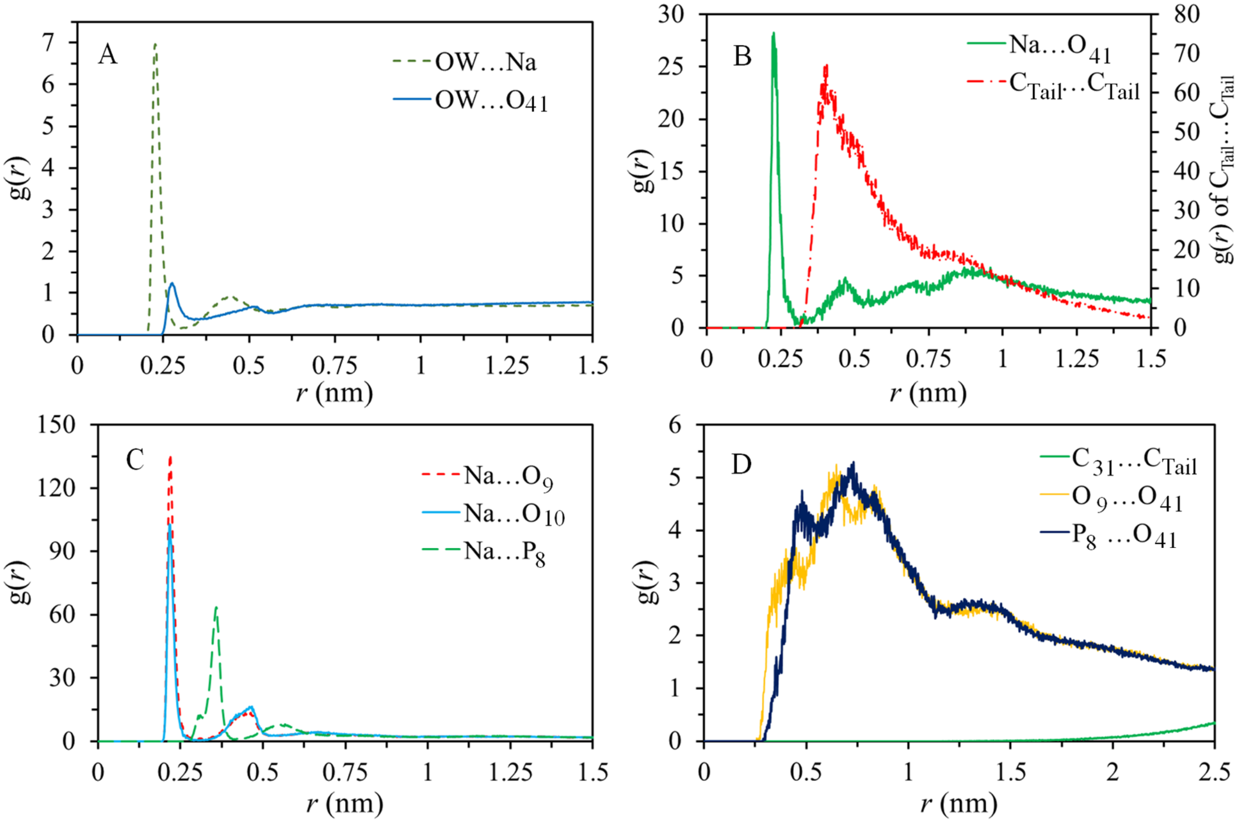

Fig. S18 Radial distribution functions of (A) OW with cation and anion of SDS (B) cation with anion of SDS and tail of SDS (C) cation of SDS with O_9, O_{10} and P_8 of POPC and (D) tail of SDS with tail of POPC and headgrups of POPC with anion of SDS.

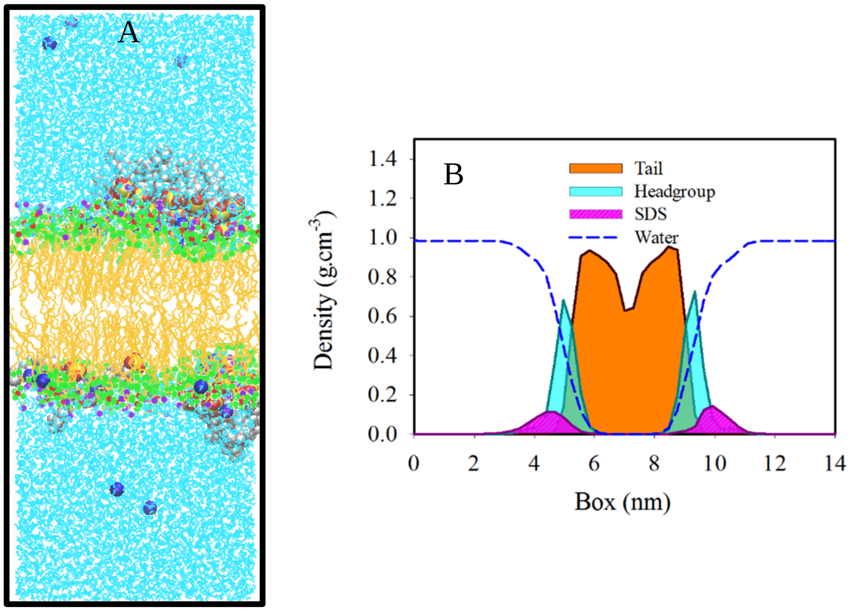

Fig. S19 (A) Snapshot of the bilayer system during MD simulations of SDS in DPPC/water after 50 ns of simulation. (B) Total density profiles of DPPC/SDS/water.

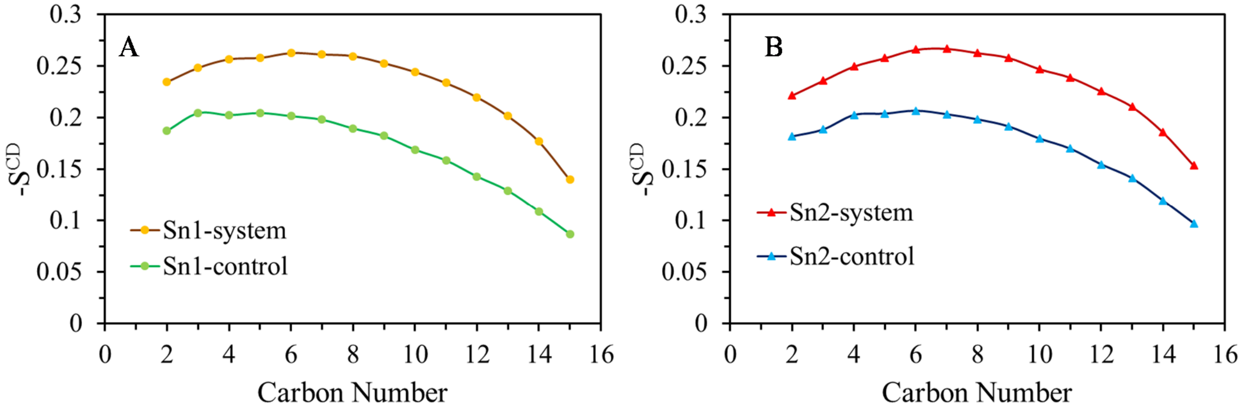

Fig. S20 (A) Average values of the simulated S^{CD} in terms of the sn-1 (B) and sn-2 chains of lipid molecules in the DPPC model membrane over the 50 ns of the simulation for SDS system. The calculated values of the control system are also shown.

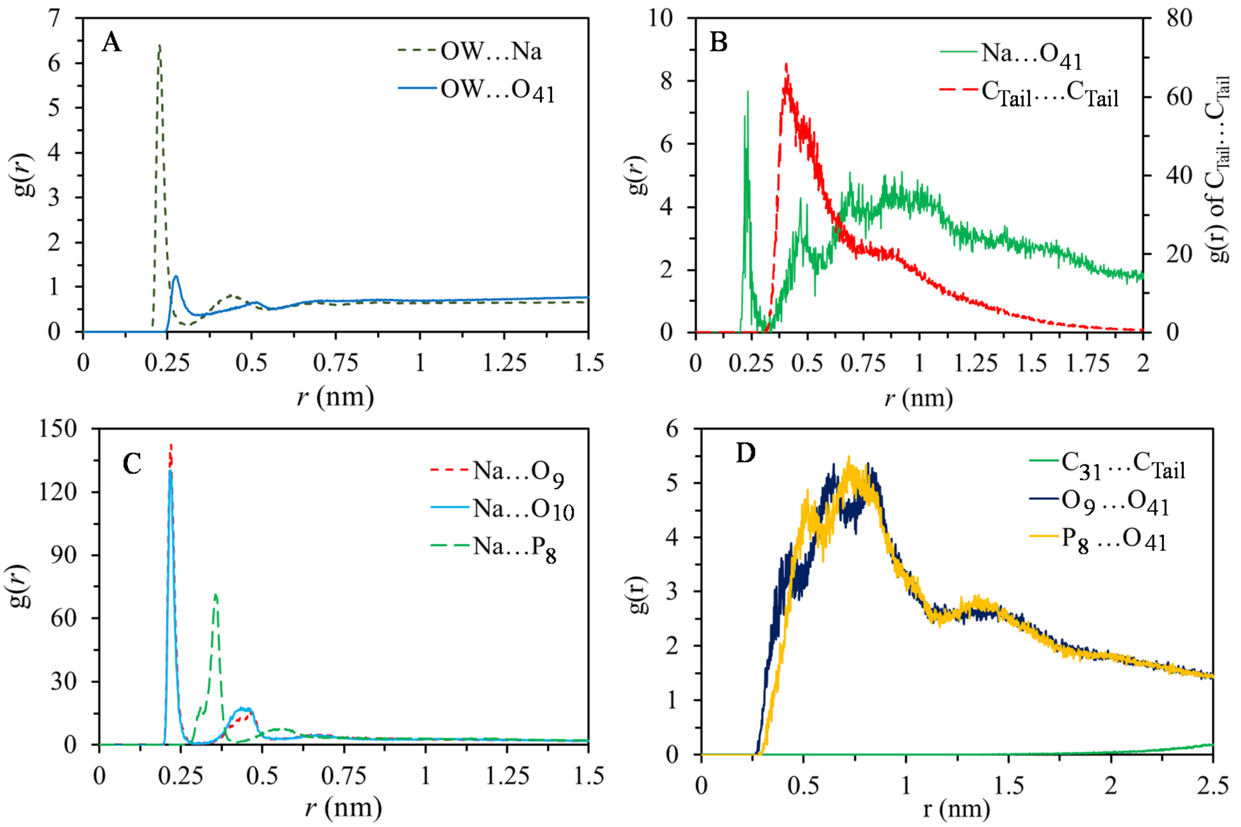

Fig. S21 Radial distribution functions of (A) OW with cation and anion of SDS (B) cation with anion of SDS and tail of SDS (C) cation of SDS with O_9, O_{10} and P_8 of DPPC and (D tail of SDS with tail of DPPC and headgrups of DPPC with anion of SDS.

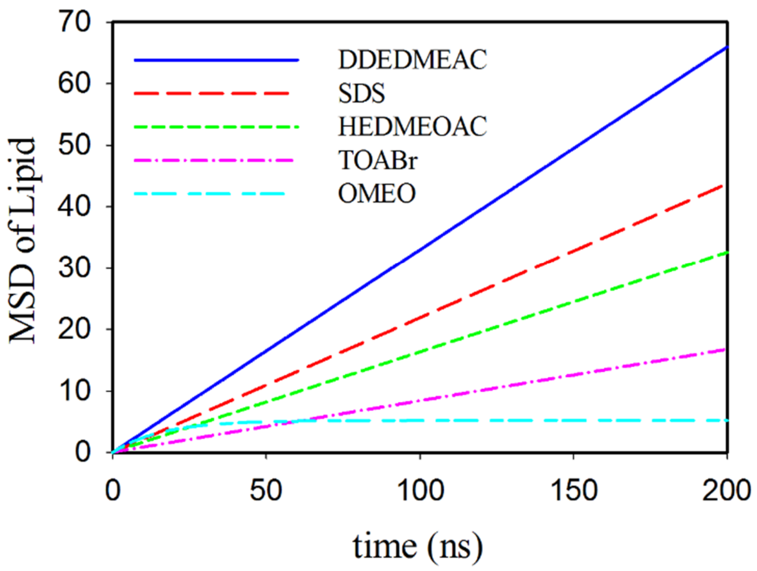

Fig. S22 Mean-squared displacements of P8 atoms of the phospholipid bilayer in the presence of various surfactants.

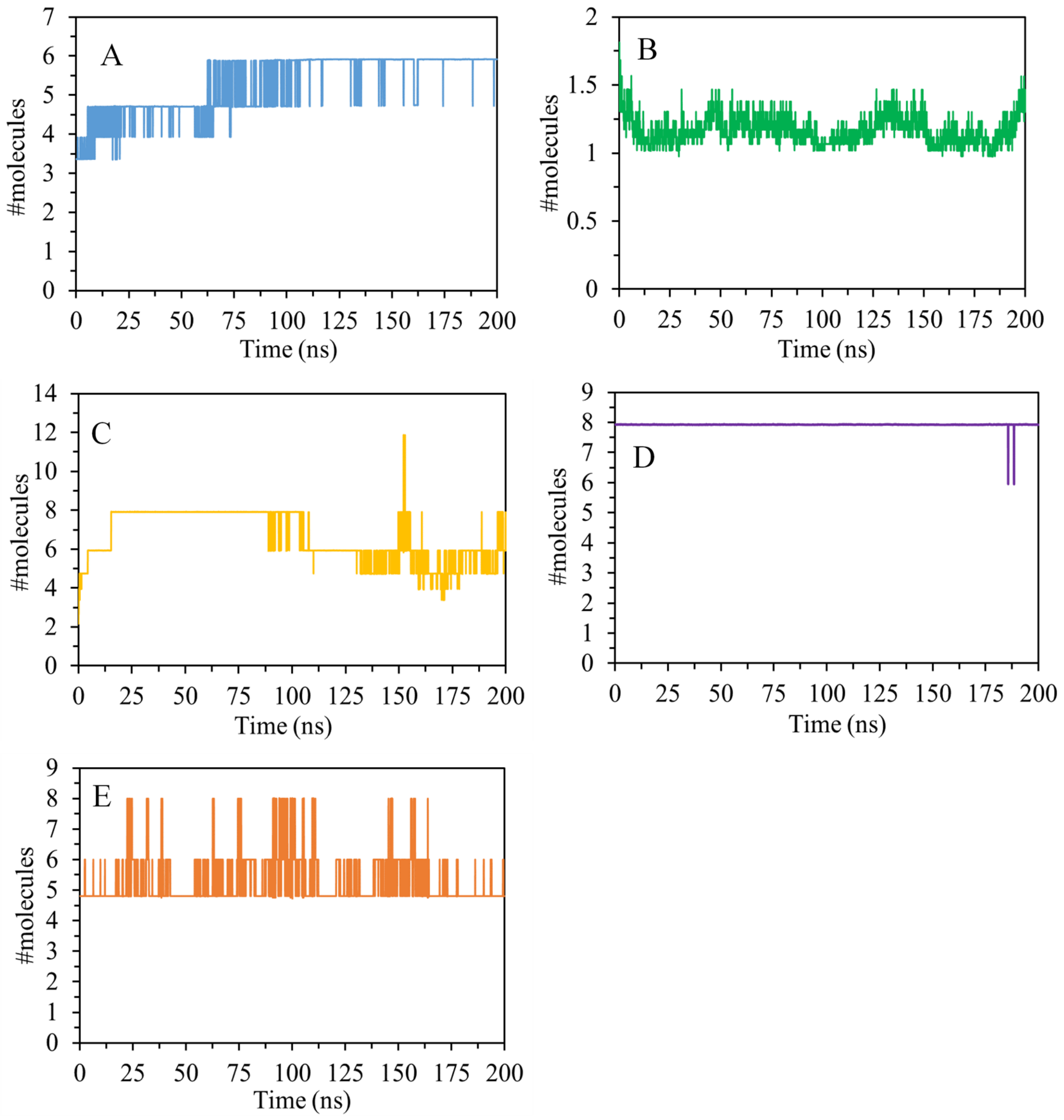

Fig. S23 Average surfactants cluster sizes from simulations of 24 molecules of (A) SDS (B) HEDMOAC (C) DDEDMEA (D) TOABr (E) OMEO in interface between water and DPPC.

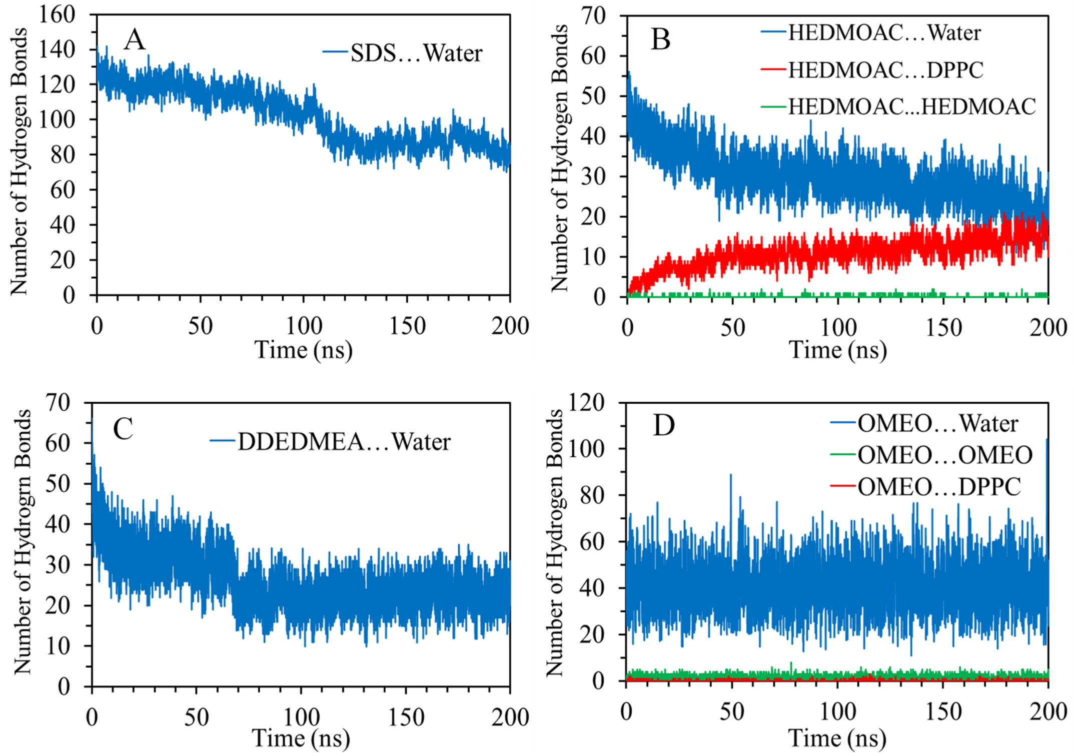

Fig. S24 Number of hydrogen bonds between (A) SDS…Water, (B) HEDMOAC…Water, HEDMOAC…DPPC and HEDMOAC…HEDMOAC, (C) DDEDMEAC…Water and (D) OMEO…Water, OMEO…DPPC and OMEO…OMEO in the systems of surfactant at the water/DPPC interface after 200ns of simulation.

Table S1. *Ab initio* calculated partial atomic charges of SDS.

atom	*q/e* B3LYP NBO	atom	*q/e* B3LYP NBO	atom	*q/e* B3LYP NBO	atom	*q/e* B3LYP NBO	atom	*q/e* B3LYP NBO
C1	-0.205	C10	-0.060	H19	0.036	H28	-0.037	H37	-0.011
C2	0.214	C11	0.056	H20	0.035	H29	-0.033	H38	-0.011
C3	0.047	C12	0.341	H21	-0.051	H30	-0.032	H39	-0.013
C4	-0.035	O13	-0.574	H22	-0.051	H31	-0.018	H40	-0.002
C5	0.109	S14	1.606	H23	-0.022	H32	-0.016	H41	-0.009
C6	0.085	O15	-0.688	H24	-0.023	H33	-0.024	H42	-0.021
C7	-0.014	O16	-0.804	H25	-0.010	H34	-0.028	NA43	0.956
C8	0.069	O17	-0.802	H26	-0.013	H35	-0.047		
C9	0.139	H18	0.043	H27	-0.038	H36	-0.044		

Table S2. *Ab initio* calculated partial atomic charges of TOABr.

atom	q/e B3LYP NBO	atom	q/e B3LYP NBO	atom	q/e B3LYP NBO	atom	q/e B3LYP NBO	atom	q/e B3LYP NBO
N1	-0.364	C22	-0.378	H43	0.189	H64	0.191	H85	0.21
C2	-0.187	C23	-0.379	H44	0.186	H65	0.199	H86	0.206
C3	-0.403	C24	-0.383	H45	0.189	H66	0.194	H87	0.205
C4	-0.377	C25	-0.573	H46	0.19	H67	0.191	H88	0.211
C5	-0.374	C26	-0.153	H47	0.188	H68	0.283	H89	0.193
C6	-0.378	C27	-0.409	H48	0.201	H69	0.203	H90	0.192
C7	-0.379	C28	-0.374	H49	0.192	H70	0.188	H91	0.192
C8	-0.383	C29	-0.376	H50	0.193	H71	0.207	H92	0.192
C9	-0.573	C30	-0.377	H51	0.193	H72	0.212	H93	0.19
C10	-0.188	C31	-0.379	H52	0.278	H73	0.192	H94	0.19
C11	-0.415	C32	-0.383	H53	0.225	H74	0.185	H95	0.189
C12	-0.378	C33	-0.573	H54	0.19	H75	0.19	H96	0.189
C13	-0.374	H34	0.281	H55	0.182	H76	0.195	H97	0.188
C14	-0.378	H35	0.202	H56	0.221	H77	0.19	H98	0.188
C15	-0.379	H36	0.192	H57	0.198	H78	0.186	H99	0.194
C16	-0.383	H37	0.202	H58	0.182	H79	0.188	H100	0.194
C17	-0.572	H38	0.213	H59	0.186	H80	0.19	H101	0.201
C18	-0.198	H39	0.191	H60	0.197	H81	0.188	BR102	-0.905
C19	-0.407	H40	0.186	H61	0.191	H82	0.201		
C20	-0.377	H41	0.191	H62	0.185	H83	0.192		
C21	-0.374	H42	0.195	H63	0.186	H84	0.193		

Table S3. *Ab initio* calculated partial atomic charges of DDEDMEAC.

atom	q/e B3LYP NBO	atom	q/e B3LYP NBO	atom	q/e B3LYP NBO	atom	q/e B3LYP NBO	atom	q/e B3LYP NBO
C1	-0.683	H18	0.229	C35	-0.311	C52	0.747	H69	0.228
H2	0.234	H19	0.239	H36	0.255	C53	-0.587	H70	0.221
H3	0.228	C20	-0.470	H37	0.310	H54	0.267	C71	-0.458
H4	0.226	H21	0.243	C38	-0.484	H55	0.286	H72	0.228
C5	-0.464	H22	0.238	H39	0.298	C56	-0.467	H73	0.229
H6	0.236	C23	-0.452	H40	0.235	H57	0.243	C74	-0.465
H7	0.231	H24	0.250	H41	0.227	H58	0.248	H75	0.230
C8	-0.462	H25	0.232	C42	-0.482	C59	-0.463	H76	0.231
H9	0.226	C26	-0.567	H43	0.239	H60	0.239	N77	-0.300
H10	0.229	H27	0.268	H44	0.228	H61	0.227	O78	-0.518
C11	-0.462	H28	0.271	H45	0.295	C62	-0.469	O79	-0.506
H12	0.232	C29	0.746	C46	-0.299	H63	0.235	C80	-0.680
H13	0.234	O30	-0.552	H47	0.274	H64	0.236	H81	0.227
C14	-0.463	O31	-0.488	H48	0.258	C65	-0.461	H82	0.233

H15	0.235	C32	-0.193	C49	-0.182	H66	0.234	H83	0.227
H16	0.227	H33	0.258	H50	0.240	H67	0.229	Cl84	-0.822
C17	-0.465	H34	0.250	H51	0.262	C68	-0.454		

Table S4. *Ab initio* calculated partial atomic charges of HEDMOAC.

atom	*q*/*e* B3LYP NBO	atom	*q*/*e* B3LYP NBO	atom	*q*/*e* B3LYP NBO	atom	*q*/*e* B3LYP NBO	atom	*q*/*e* B3LYP NBO
N1	-0.341	C10	-0.497	H19	0.235	H28	0.242	H37	0.271
C2	-0.495	C11	-0.278	H20	0.231	H29	0.233	H38	0.267
C3	-0.471	C12	-0.141	H21	0.238	H30	0.235	H39	0.220
C4	-0.465	O13	-0.785	H22	0.241	H31	0.241	H40	0.220
C5	-0.469	H14	0.313	H23	0.234	H32	0.246	H41	0.517
C6	-0.468	H15	0.244	H24	0.231	H33	0.312	Cl42	-0.869
C7	-0.478	H16	0.234	H25	0.234	H34	0.242		
C8	-0.697	H17	0.252	H26	0.237	H35	0.312		
C9	-0.495	H18	0.264	H27	0.234	H36	0.244		

Table S5. *Ab initio* calculated partial atomic charges of OMEO.

atom	*q*/*e* B3LYP NBO	atom	*q*/*e* B3LYP NBO	atom	*q*/*e* B3LYP NBO	atom	*q*/*e* B3LYP NBO
O1	-0.787	C11	-0.467	H21	0.173	H31	0.233
C2	-0.135	C12	-0.468	H22	0.205	H32	0.233
C3	-0.135	C13	-0.477	H23	0.205	H33	0.233
O4	-0.595	C14	-0.697	H24	0.247	H34	0.234
C5	0.230	H15	0.505	H25	0.247	H35	0.234
O6	-0.592	H16	0.212	H26	0.234	H36	0.241
C7	-0.110	H17	0.212	H27	0.234	H37	0.234
C8	-0.491	H18	0.215	H28	0.235	H38	0.234
C9	-0.470	H19	0.215	H29	0.235		
C10	-0.466	H20	0.173	H30	0.233		

Table S6. Self-diffusion coefficients ($1\times10^{-10}\ m^2/_s$) of surfactants candidate molecules calculated by *MSD*–time curves (β values in parentheses).

Surfactant	Diffusion Coefficient of Anion	Surfactant	Diffusion Coefficient of Cation
SDS	2.10±0.25 (0.78)	SDS	4.19±0.30 (0.75)
HEDMOAC	26.13±0.65 (0.82)	HEDMOAC	6.26±0.34 (0.84)
DDEDMEAC	20.70±0.41 (0.74)	DDEDMEAC	3.84 ±0.18 (0.68)
TOABr	16.95±0.52 (0.78)	TOABr	4.78±0.23 (0.76)
OMEO	5.03±0.35 (0.82)		

Table S7. Average cluster Size [molecules] of surfactant molecules at water/bilayer interface.

Surfactant	Ave. surfactant cluster size [interface]	Ave. surfactant cluster size [bulk]
SDS	5.4±0.8	3.3±1.2
TOABr	8.0±0.0	24.0±0.0
DDEDMEA	6.6±1.2	20.0±0.1
HEDMOAC	1.2±0.1	4.4±0.1
OMEO	5.8±0.7	9.0±0.1
